# Predicting Metabolic Adaptation Under Dynamic Substrate Conditions Using a Resource-Dependent Kinetic Model: A Case Study Using *Saccharomyces cerevisiae*

**DOI:** 10.3389/fmolb.2022.863470

**Published:** 2022-05-16

**Authors:** K. J. A. Verhagen, S. A. Eerden, B. J. Sikkema, S. A. Wahl

**Affiliations:** ^1^ Department of Biotechnology, Delft University of Technology, Delft, Netherlands; ^2^ Lehrstuhl für Bioverfahrenstechnik, FAU Erlangen-Nürnberg, Erlangen, Germany

**Keywords:** proteome adaptation, kinetic modeling, *Saccharomyces cerevisiae*, Crabtree effect, resource allocation, dynamic conditions, feast/famine

## Abstract

Exposed to changes in their environment, microorganisms will adapt their phenotype, including metabolism, to ensure survival. To understand the adaptation principles, resource allocation-based approaches were successfully applied to predict an optimal proteome allocation under (quasi) steady-state conditions. Nevertheless, for a general, dynamic environment, enzyme kinetics will have to be taken into account which was not included in the linear resource allocation models. To this end, a resource-dependent kinetic model was developed and applied to the model organism *Saccharomyces cerevisiae* by combining published kinetic models and calibrating the model parameters to published proteomics and fluxomics datasets. Using this approach, we were able to predict specific proteomes at different dilution rates under chemostat conditions. Interestingly, the approach suggests that the occurrence of aerobic fermentation (Crabtree effect) in *S. cerevisiae* is not caused by space limitation in the total proteome but rather an effect of constraints on the mitochondria. When exposing the approach to repetitive, dynamic substrate conditions, the proteome space was allocated differently. Less space was predicted to be available for non-essential enzymes (reserve space). This could indicate that the perceived “overcapacity” present in experimentally measured proteomes may very likely serve a purpose in increasing the robustness of a cell to dynamic conditions, especially an increase of proteome space for the growth reaction as well as of the trehalose cycle that was shown to be essential in providing robustness upon stronger substrate perturbations. The model predictions of proteome adaptation to dynamic conditions were additionally evaluated against respective experimentally measured proteomes, which highlighted the model’s ability to accurately predict major proteome adaptation trends. This proof of principle for the approach can be extended to production organisms and applied for both understanding metabolic adaptation and improving industrial process design.

## Introduction

The ability of microorganisms to adapt to changing extracellular environmental conditions is essential for their survival and leads to metabolic robustness and competitive fitness (Gerosa and Sauer, 2011; Chubukov et al., 2014). Depending on the environmental conditions, different metabolic functions and/or flux distributions are needed that require a different proteome composition (Litsios et al., 2018). The proteome adaption is triggered by not yet fully unraveled protein signaling cascades and further mechanisms (Zhao et al., 2016). An intuitive example of this adaption is described for *Saccharomyces cerevisiae* (*S. cerevisiae*) when shifting from growth under minimal to rich medium conditions; cells grown under rich nutrient conditions require a significantly smaller proteome fraction for biosynthesis genes (de Godoy et al., 2008; Nagaraj et al., 2012; Liebermeister et al., 2014) than cells grown in the mineral medium, in which amino acids and other biomass precursors are not present but have to be synthesized from glucose.

On the other hand, next to optimization of proteome resources, cells do maintain metabolic fitness and/or robustness (Basan, 2018), especially under substrate limiting conditions cells seem to invest in proteins that may not be required yet, for example, to quickly utilize alternative substrates without delays in growth (Dekel and Alon, 2005). However, any additional increase in protein abundance also results in higher costs due to occupation of ribosomes, resource consumption, and potentially additional protein misfolding. Different hypotheses have been formulated and respective models were developed to understand the optimization and trade-offs.

Constraint-based modeling approaches are essential to analyze putative properties of metabolic networks. The well-established and frequently used method for the analysis of (large genome-scale) metabolic networks is flux balance analysis (FBA) (Varma and Palsson, 1994; Orth et al., 2010). This method calculates feasible solutions under steady-state conditions, depending on a defined objective function (biomass or ATP maximization) (Schuetz et al., 2007). However, this method cannot be applied to dynamic cultivation conditions and does not consider gene regulation or protein expression. To overcome these limitations, dynamic flux balance analysis (dFBA) was developed to maximize biomass growth over time, with changing extracellular conditions (Mahadevan et al., 2002). To include the synthesis costs of proteins and ribosomes, resource balance analysis (RBA) was developed, allowing for the prediction of the optimal allocation of intracellular resources for steady-state growth (Goelzer et al., 2015). Looking at cellular behavior in terms of resource allocation has also been used to explain overflow metabolism (Basan et al., 2015; Nilsson and Nielsen, 2016). In this paradoxical phenomenon, cells use catabolic pathways with low ATP yields per substrate such as alcoholic fermentation when growing at high growth rates, even when a high-yield pathway such as respiration is available. Following the current hypothesis, the answer is that these fermentative pathways are much cheaper in terms of proteome space cost, meaning that the ATP production rate per protein mass is larger (Nilsson and Nielsen, 2016).

Combining approaches from both dFBA and RBA leads to conditional FBA (cFBA) (Rügen et al., 2015; Reimers et al., 2017), which combined both temporal changes in the extracellular environment with constraints on intracellular resource allocation. These powerful tools are able to reproduce and predict metabolic phenotypes beyond steady-state conditions and extend our understanding of microbial physiology. Nevertheless, short-term dynamics require yet another mechanism: kinetics instead of a quasi-steady state of the intracellular metabolites to capture the rapid intracellular changes of metabolites as well as kinetic regulation.

Experimentally, *S. cerevisiae* cultures have a different metabolic response to substrate perturbations depending on the cultivation condition, especially cells cultured under repetitive dynamic substrate conditions, the so-called “feast/famine” regime showed a different response compared to cultures grown under steady-state limitations (Suarez-Mendez et al., 2014). Ethanol production after a substrate pulse was observed for cultures originating from a chemostat (Wu et al., 2006), while no ethanol was observed for cells under a repetitive excess/limitation regime (Suarez-Mendez, 2015). Furthermore, the intracellular response to substrate excess has significantly different properties: while the ATP concentration dropped after a pulse originating from a chemostat culture (Wu et al., 2006), a rise was observed for a feast/famine culture. Moreover, the biomass yield of a feast/famine culture was lower than that of a chemostat culture. Last, chemostat-grown cells showed short- and long-term accumulation of glycolytic intermediates after a substrate pulse, while this was not observed for feast/famine cultures. Storage synthesis and degradation leads to “wasting” of ATP (futile cycle) which was shown to rescue cellular metabolism, that is, balance pathway capacities in case of sudden perturbations (van Heerden et al., 2014).

The observed differential metabolic response implies an adaptation during the prior dynamic growth condition. Similar differences in adaptations have been observed earlier, for example, the lag phase before exponential growth (Brejning and Jespersen, 2002; Jõers and Tenson, 2016), upon a change in the substrate (Chu and Barnes, 2016), and in the period just after switching to a different dilution rate in a chemostat (Abulesz and Lyberatos, 1989).

There are three levels of metabolic regulation commonly assumed to be dominant (Wegner et al., 2015): 1) allosteric regulation, in which enzyme activity is modified by non-covalent binding with other molecules. The response time of this type of regulation is almost instant (Pincus et al., 2017), and it is often used for local fine-tuning in metabolism, and thus it is unlikely to cause this adaptation effect. 2) Post-translational modifications (PTMs), in which enzyme activity is altered by the addition of covalent attachments. The timescale of this response is a matter of seconds to minutes (Karim et al., 2014), and it is often part of short-term responses to stress situations (e.g., sudden changes in the environment). 3) Translational regulation, which influences the composition of the proteome. This regulation has a response time of hours (Cohen et al., 2008), which is in the same order of magnitude as the generation time, and thus the choices made at this level are important for the long-term strategy. It is also considered the most expensive regulatory level: degradation and synthesis of proteins requires significant amounts of ATP.

Recent studies have shown that the amount of protein in a cell is limited due to macromolecular crowding, the kinetics of protein synthesis, and degradation (Vazquez et al., 2008; Molenaar et al., 2009). When all the proteome space is occupied, increasing the concentration of one protein is only possible at the cost of another (Pareto Frontier) (Mori et al., 2019).

We were curious to study the impact of short-term vs. long-term adaptations to substrate perturbations encountered in natural and laboratory environments. Therefore, we developed a resource-dependent kinetic model and exposed this to different dynamic environments to evaluate the impact of the allocation of proteins in the cellular proteome on the metabolic fitness of a yeast cell under short-term extracellular substrate dynamics.

## Materials and Methods

### Proteome-Dependent Kinetic Yeast Model

The proteome-dependent kinetic yeast model is based on a system of ordinary differential equations (ODEs) that describe the mass balances of all intra- and extra-cellular metabolites. This system of ODEs is solved with the ode15s function in MATLAB 2020b, for which the absolute tolerance is set to 1e-4, and all variables are constrained to be higher than zero with the “nonnegative” setting. A detailed description of the final proteome-dependent kinetic yeast model used is given in Supplementary Material S1.

To predict which proteome composition is the most competitive for defined environmental conditions, a Monte Carlo approach is used. The metabolic behavior of 1,000 random proteomes, generated around a seed proteome, is compared based on an objective function. Under steady-state conditions, the minimization of the residual substrate concentration was used as an objective function. Under dynamic conditions, the minimization of a time-weighted average substrate concentration was used, to promote fast consumption of the available substrate, therefore selecting for competitive proteomes:

$$\;\frac{\int_{0}^{t_{cycle}}c_{s}\cdot t\;dt}{\int_{0}^{t_{cycle}}tdt}.$$

Subsequently, it is determined whether the solution is balanced. If the objective function is optimized and the solution is balanced, the objective function and the seed proteome are updated. In the next iteration, the proteome is then generated around this new seed proteome, with a maximum deviation of 25% per sector.

### Proteome Allocation to Model Sectors

All proteins from experimental datasets are sorted in the same nine protein sectors that are used in the model, to allow for a direct comparison of the experimental proteomes and the optimized proteomes. The proteins are categorized per sector based on either the protein name or the description in the KEGG database (Goffeau et al., 1996; Kanehisa et al., 2016) (Supplementary Material S7). The whole dataset is sorted with the MATLAB 2020b functions “strcmp” and “contains,” which are used to search the dataset for specific names or keywords to group the proteins.

### Parameter Optimization

The proteome cost parameters are estimated by optimization with the MATLAB 2020b function fmincon. For all parameter optimizations, a multi-start approach is used. This approach minimizes the risk of reaching a local minimum in the solution space by starting the optimization from different initial guesses. The tolerance of the function is set to 1e-12 for all optimizations. For the estimation of the k_cat_ parameters, the difference between the experimental and simulated fluxes is minimized. Additional weight in the objective function was applied for the growth rate, as k_cat_ parameters have to be rejected if the maximum growth rate is not reached.

### Overcapacity Simulations

The amount of overcapacity in the yeast proteome is determined by introducing a 10th protein sector. This new protein sector does not have a function for the cells, and hence, only takes up space in the proteome. Therefore, the fraction of the proteome that can be allocated into the extra sector without altering the metabolic fluxes is defined as overcapacity. The overcapacity is estimated for each sector separately, to minimize the changes in each step. The sectors are sorted in a decreasing order and then optimized for overcapacity in this order. The amount of overcapacity in each sector is determined in a step-wise approach. Per iteration, one percent of the specific protein sector is removed and allocated into the extra sector. Subsequently, the fluxes of the adapted proteome are compared to the reference fluxes, and only if the change in the fluxes remains within the boundaries, the seed proteome is updated. This new seed proteome is then used for the next iteration, in which the sector size is again decreased by 1%. By decreasing the sector size by 1% of the current size, the step size is reduced with each iteration. If the flux profile deviates more than the threshold value, the adapted proteome allocation is rejected. The fluxes are evaluated based on the following criterion: the average value of the uptake and growth fluxes should not deviate more than 1% from the reference flux, to ensure that the same substrate uptake and growth rates are achieved.

## Results

### Construction of a Proteome-Dependent Kinetic Model

**FIGURE 1:**
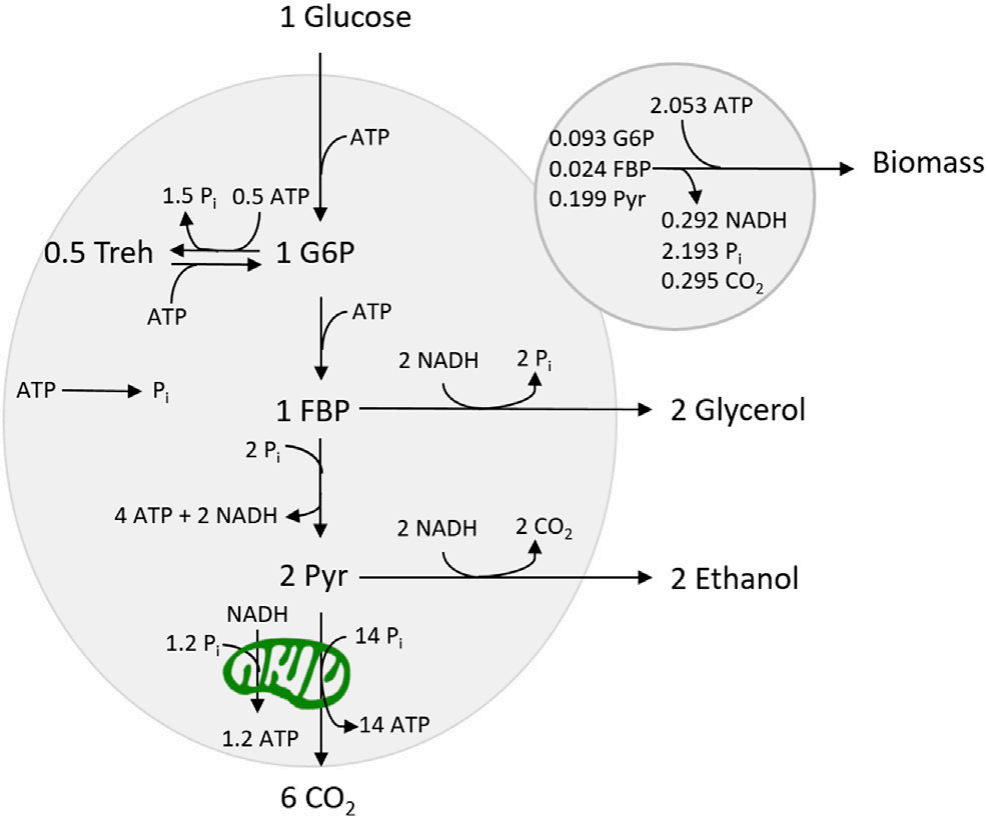
Map showing the metabolic network used in this model.

We wanted to construct a proteome-dependent kinetic model, which was small, but still able to reproduce the main phenotypes observed for *S. cerevisiae*. Furthermore, it should be calibrated with available experimental data. We constructed the model based on the kinetic model of yeast glycolysis (Teusink et al., 2000) which we extended with reactions for the trehalose cycle and respiration pathway as well as a growth reaction (see Figure 1). Each (lumped) reaction has been associated with a proteome fraction resulting in a proteome-dependent kinetic model of yeast central carbon metabolism and growth.

The Embden–Meyerhof glycolytic pathway has been implemented as three lumped reactions (uptake, upper, and lower glycolysis) with three intermediates: G6P, FBP, and pyruvate. The stoichiometry of the growth reaction was based on Suarez-Mendez et al. (2016). The NADPH requirement was assumed to be met by using the pentose phosphate pathway, which in sum (together with PGI) converts one G6P to six CO_2_ and 12 NADPH. The required NADPH flux was balanced by a respective consumption of G6P. The ATP demand for growth has been derived from Della-Bianca et al. (2014) taking into account that the demand was expressed as catabolized glucose amounts. Furthermore, the trehalose cycle was included as two lumped reactions, based on an existing kinetic model of the trehalose cycle (Smallbone et al., 2011) (see Supplementary Material S1 for details).

Due to a lack of kinetic models of yeast TCA cycle and oxidative phosphorylation, the two respiratory reactions (from cytosolic NADH and pyruvate, v_NDE_, and v_TCA_, respectively) have been implemented using general Michaelis–Menten kinetics. However, the two reactions are interdependent—both connect to the electron transport chain—and consequently, the rate is determined by the same proteome fraction. A maximum value for the rate of the two reactions combined is defined, reflecting the capacity in the electron transport chain, limited by the provided proteome sector size (see Supplementary Material S1).

The biomass reaction contains many complex reactions, and the kinetics of the full process currently cannot be derived from basic principles. Therefore, a holistic approach based on experimental observations was chosen, i.e., the growth rate has been found to correlate with the energy charge (Boer et al., 2010). Here, the growth rate is described by a sigmoid function that is the most sensitive within the range of an energy charge between 0.7 and 0.9 as observed for growing cells (Boer et al., 2010).

### Calibration of Model Parameters Using Available Experimental Data

The specific activity for the defined pathways has a major impact on model predictions. To obtain realistic values, the specific enzyme activities (k_cat_) were estimated from experimental omics datasets. In the proposed model, the k_cat,i_ for each reaction i is defined as the maximum reaction rate per fraction of proteome (mol/Cmol_x_/h), where 100% proteome reflects 500 mg protein per g_X_ (Ertugay and Hamamci, 1997). Hence, the maximum rate of the reaction i ( $V_{max,i}$ ) with a given sector fraction $\varphi_{i}\;$ is:

$$V_{\max,i}=\;\varphi_{i}\cdot k_{\mathrm{cat},i}.$$

From this, $\vec{c}\;$ enzymatic rate $V_{i}$ is calculated by multiplying the $V_{\max,i}$ with the function $f_{i}\left(\vec{c}\;\right)$ describing the effects on the enzymatic rate due to substrate and product concentrations as well as effects by allosteric activators and inhibitors (see Supplementary Material S1 for specification of $f_{i}\left(\vec{c}\;\right)$ for each reaction):

$$V_{i}=\;V_{\max,i}\cdot f_{i}\left(\vec{c}\;\right).$$

The k_cat_ parameters have been estimated by combining the proteome and fluxome measurements under batch conditions. The proteome fractions were taken from de Godoy et al. (2008) using *S. cerevisiae* grown under batch conditions with a defined glucose minimal medium and aligned according to the protein classification in the KEGG database. Specifically, grouping all proteins with the KEGG BRITE label “Genetic Information Processing” and all proteins with the “Metabolism” label that were not classified as “Central Carbon Metabolism” or “Energy Metabolism” being assigned to the “growth protein sector,” assuming that their size is growth rate-dependent in the minimal medium. Furthermore, for the calculations, it was assumed that the whole proteome sector of cells grown under excess substrate at the maximal growth rate was used.

The corresponding flux distribution, i.e., under batch conditions was obtained from Heyland et al. (2009) with the exception of fluxes for the trehalose cycle—these were obtained from the feast/famine experiments conducted by Suarez-Mendez et al. (2017). For both trehalose synthesis and degradation, the maximum value of the flux reached in one feast/famine cycle was used, which was 5.10⋅10^−3^ mol/Cmol_X_/h for trehalose synthesis and 4.09·10^−3^ mol/Cmol_X_/h for the degradation of trehalose. The k_cat_ value for maintenance was set to 0.0155 mol/Cmol_X_/h, which is the maintenance requirement measured at near-zero growth rates (Vos et al., 2016).

To obtain the k_cat_ parameters, parameter optimization was performed, estimating the parameters which produced the smallest deviation between the simulated and experimental fluxes (Heyland et al., 2009), using the batch proteome composition taken from de Godoy et al. (2008) (see Supplementary Material S2 for details). Using this approach, the proteome-dependent kinetic model was able to largely reproduce the experimental flux distribution (Table 1), and this k_cat_ calibration was used in all further calculations.

**TABLE 1:** Comparison of the predicted fluxes of a chemostat experiment at a dilution rate of 0.4 h^−1^ with the experimental flux distribution of Heyland et al. (2009). Upt, uptake; UGlc, upper glycolysis; LGlc, lower glycolysis; Ferm, fermentation; Esnk, electron sink/glycerol pathway; Resp, respiration; Trsn, trehalose synthesis; Trdg, trehalose degradation; Grwt, growth.

	Upt	Uglc	LGlc	Ferm	Esnk	Resp	Trsn	Trdg	Grwt
Predicted flux (mol/Cmol_x_/h)	0.504	0.4669	0.8251	0.7391	0.0898	0.0719	0.0072	0.0072	0.4
Experimental flux (mol/Cmol_x_/h)	0.4753	0.4373	0.8745	0.7272	0.0428	0.0808	0.0051	0.0041	0.4
Deviation	+6%	+7%	-6%	+2%	+110%	-11%	+41%	+76%	0%

### Prediction of the Steady-State Growth Phenotype Under Carbon-Limited Steady-State Conditions

*S. cerevisiae* is a Crabtree-positive yeast, and thus fermentation is observed next to oxidative phosphorylation at substrate uptake rates above an observed “critical” rate (Barford and Hall, 1979). The ability of the model to reproduce the Crabtree effect is assessed by optimizing proteomes for dilution rates in the range from 0.05 h^−1^ to 0.4 h^−1^. The proteome optimization was started at the dilution rate of 0.4 h^−1^ using the experimental batch proteome as a starting value. The most competitive proteome out of 1,000 randomly generated proteome allocations was selected using minimization of the residual substrate concentration as an objective function. Subsequently, this procedure was repeated for the next lower dilution rate. The optimal proteome allocation of the previous dilution rate was used as a starting value. To validate the model, the predicted fluxes and metabolite concentrations were compared with a flux and metabolome dataset (Suarez-Mendez et al., 2016) at different dilution rates under chemostat conditions. This comparison of predicted and measured fluxes and metabolite concentrations can be found in Supplementary Material S3 and in Supplementary Figures S2, S3, respectively.

**FIGURE 2:**
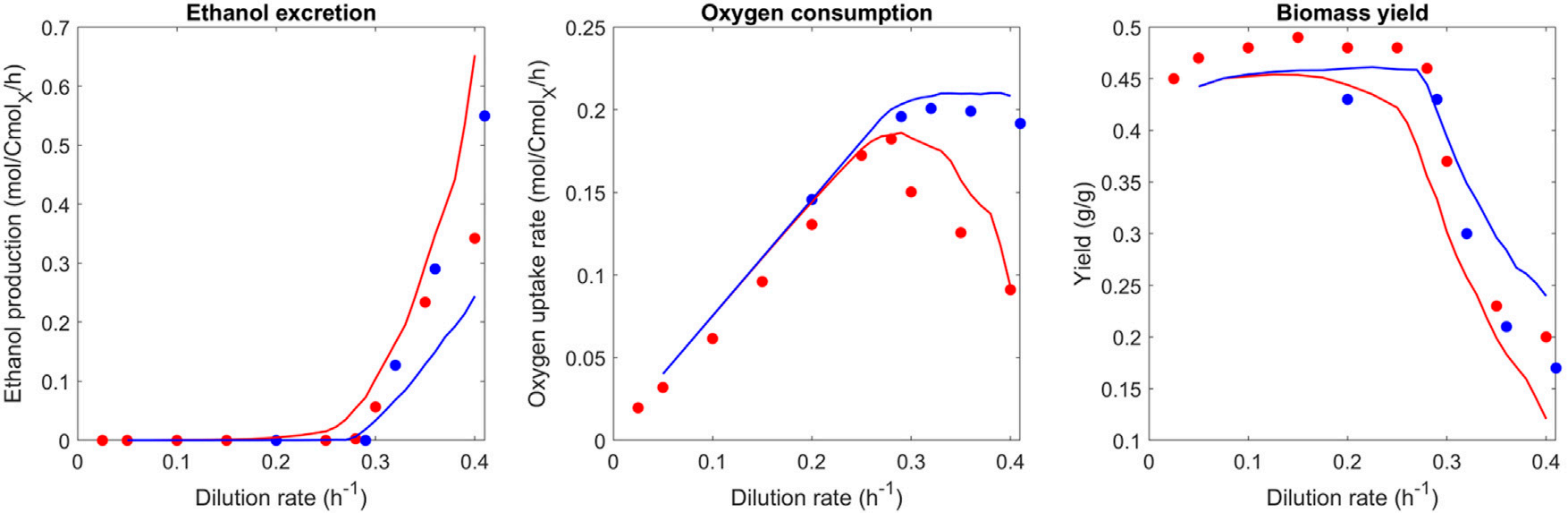
Comparison of predicted and observed phenotypic rates (ethanol excretion, oxygen uptake rate, and biomass yield) at different dilution rates. Blue represents the best proteome out of 1,000 randomly generated proteomes; red represents the best proteome out of 100 randomly generated proteomes (limited evolution with adaptation from the batch proteome). For the experimental data similarly–red represents data from Van Hoek et al. (1998) (seven generations at steady-state starting from batch), and blue represents a respiration-adapted culture (Rieger et al., 1983).

The experimental data for ethanol production and oxygen consumption in Figure 2 show that the ethanol production starts at a dilution rate of 0.28 h^−1^ (Rieger et al., 1983; Van Hoek et al., 1998). Above this critical dilution rate, the oxygen consumption rate decreases, while ethanol production keeps increasing. Ethanol production is first predicted by the model for a dilution rate of 0.25 h^−1^, which is a lower rate than the experimental data. Furthermore, there is no decrease in the oxygen consumption rate above a dilution rate of 0.28 h^−1^ for the optimized proteomes, which was observed in experimental studies (Van Hoek et al., 1998). From the model, this can be explained by the proteome-specific ATP production “cost”: Respiration has a high yield compared to fermentation (Table 2.). Hence, reducing the size of the respiration proteome sector will not be predicted by the model as it is not beneficial. The predicted plateau originates from a constraint that was introduced manually (12% of the proteome for respiration) to reflect the maximum oxygen consumption rate measured by Rieger et al. (1983) after long-term evolution. The continuous increase in the ethanol production rate can then be explained by the increasing need for ATP with an increasing growth rate while respiration is at its maximum.

**TABLE 2:** Comparison of the proteome-specific ATP yield for fermentation and respiration obtained by Nilsson and Nielsen( 2016) and this study. Values of this study were derived from simulations performed at a growth rate of 0.4 h^−1^.

	Fermentation (mol_ATP_/g_prot_/h)	Respiration (mol_ATP_/g_prot_/h)
Nilsson and Nielsen (2016)	0.40	0.21
This study	0.18	0.20

This result conflicts with the discussed dataset of Van Hoek et al. (1998) as well as the model predictions of Nilsson and Nielsen (2016), which was partly based on this experimental dataset. This mismatch and conclusions will be discussed in more detail later. Notably, there is also experimental evidence from previous studies that the predicted plateau is reasonable. It was shown that the respiratory repression observed by Van Hoek et al. (1998) could be negated upon long-term adaptation (Barford and Hall, 1979; Rieger et al., 1983; Postma et al., 1989), and a stable maximum oxygen uptake rate above a dilution rate of 0.28 h^−1^ was found.

To test the hypothesis of short- vs. long-term evolution, the proteome optimization approach was performed with a reduced number of generated proteomes and compared to the experimental data of Van Hoek et al. (1998) (Figure 2, red line). With a high number of generated proteomes for the optimization, the experimental findings of long-term chemostats could be reproduced. From these predictions, we hypothesize that cells not exposed to long-term glucose-limited conditions did not yet reach the “optimal” proteome allocation and respective metabolic phenotype. This set number of 1,000 simulations was chosen because only very limited further optimization of the objective function was observed after this number of simulations. As such, 1,000 simulations were concluded as sufficient to reach the optimum. Work on adapted glucose-grown cultures, at which point glucose repression on respiration disappears, is cultivated for at least 50 generations at the same dilution rate (Barford and Hall, 1979). A work by Van Hoek et al. (1998) describes the Crabtree effect with its typical glucose repression of respiration, by cultivating cultures at the same dilution rate for seven generations. Therefore, a set number of 100 simulations was chosen to reflect this state of limited adaptation of the proteome from batch growth conditions.

**FIGURE 3:**
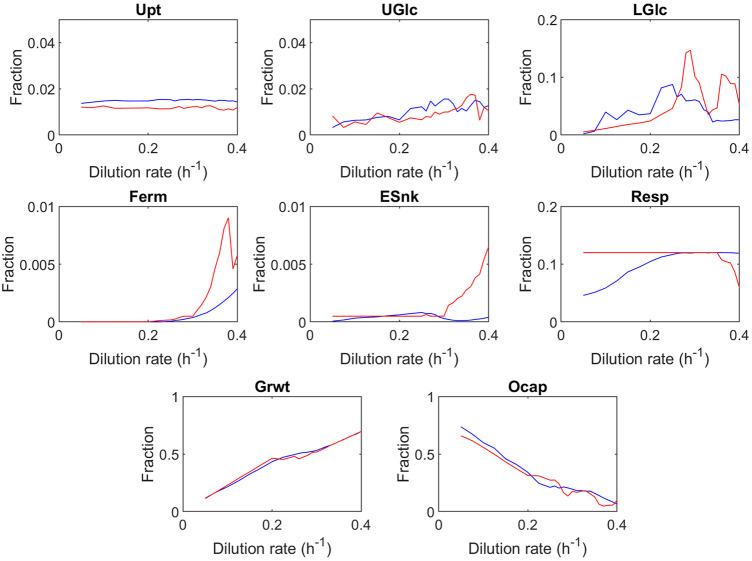
Predicted proteome fractions at steady-state as a function of the dilution rate. Blue represents the best proteome out of 1,000 randomly generated proteomes; red represents the best proteome out of 100 randomly generated proteomes. The values shown are averaged over 40 optimization runs, and the single results are displayed in the Supplementary Figure S4. Upt, uptake; UGlc, upper glycolysis; LGlc, lower glycolysis; Ferm, fermentation; Esnk, electron sink/glycerol pathway; Resp, respiration; Grwt, growth; Ocap, overcapacity. The trehalose sector was decreased to zero in all instances of the overcapacity simulation, and therefore not shown in the figure.

Looking into the global trends in the fully evolved proteome allocation at different dilution rates (Figure 3, see Supplementary Material S4 for sensitivity analysis), an increase in the dilution rate can be seen for nearly all sectors leading to the unused space (in the following called overcapacity sector, last panel). The overcapacity sector accounts for the fraction of the proteome which remains unused within the optimized proteomes. Before discussing specific trends, the high dilution rates will be highlighted. Even close to the maximal growth rate, the model predicts a small overcapacity sector. Nevertheless, please note that the batch and very high dilution rate might still have different optimization criteria; here, in the model, minimal substrate concentration was applied as the objective function. Because of the optimization approach, some robustness is required that was not further tuned as the fraction is rather small (7%) and does not change trends. Additionally, the algorithm samples from an enumerated number of randomly generated proteomes and therefore requires some buffer for robustness.

A major difference between this model and earlier studies (Nilsson and Nielsen, 2016) is that the proteome space limit is not reached at the critical growth rate (D = 0.28 h^−1^). At the critical dilution rate (D = 0.28 h^−1^), the overcapacity sector still has a significant fraction (21%). As briefly discussed earlier, Nilsson and Nielsen (2016) postulated that the Crabtree effect could be explained by the catalytic efficiency of the fermentation and respiration pathways expressed as ATP per amount of protein used in the pathway (Table 2). To estimate these catalytic efficiencies, Nilsson and Nielsen (2016) used the fluxes and specific enzyme activities for fermentation and respiration, under the assumption that all enzymes operate at half of their maximum specific activity , whereas in this model, the estimation of the catalytic efficiency is based upon the proteome and fluxome dataset, using dynamic saturation of enzymes. The estimation proposed by Nilsson and Nielsen (2016) subsequently produced a proteome composition in which the mass of all respiration proteins is 19 times larger than the protein mass of all glycolysis enzymes , while from proteome measurements it was observed that the mass of all respiration proteins is 0.3 times the size of the mass of all glycolysis proteins (de Godoy et al., 2008; Elsemman et al., 2022). This large difference in proteome allocation between glycolysis and respiration causes the catalytic efficiency of fermentation to be overestimated. The conclusion that the proteome is fully allocated after the critical growth rate is reached leads to the prediction that the “optimal” endpoint of proteome allocation is reached, which cannot explain datasets by Barford and Hall (1979); Rieger et al. (1983). Additional modeling studies by Elsemman et al. (2022) suggest that the decrease in oxygen consumption at higher growth rates observed by Van Hoek et al. (1998) is not caused by a limitation in proteome capacity but rather by a maximum rate of mitochondria biogenesis, in which long-term adaptation could overcome the described glucose repression of respiration.

### Prediction of Proteome Allocation Under Dynamic Conditions

The proteome compositions, especially at low dilution rates were characterized by a significant overcapacity sector. The kinetic proteome allocation approach could not yet answer why the cells maintained such an excess proteome. As discussed earlier, the hypothesis for a proteome overcapacity is competitiveness and robustness including dynamic environmental conditions. Overcapacity could enable faster substrate uptake rates and enable a competitive advantage and outcompete slower consuming microbes (Jannasch, 1967). Furthermore, excess capacity could enable a robust, balanced functioning of pathways such as glycolysis (van Heerden et al., 2014) under dynamic substrate conditions.

To test these hypotheses, we studied the predicted proteome allocation under different repetitive substrate-feeding regimes using the proteome-dependent kinetic model, using the minimization of the time-weighted residual substrate concentration as the objective function. With this approach, we were able to select competitive proteomes with fast substrate uptake rates. As a reference dynamic condition, an experimentally explored feeding regime was chosen, i.e., a cycle length of 400 s of which 20 s was used to feed the culture (D = 2 h^−1^), leading to the average dilution rate of D = 0.1 h^−1^ over the complete cycle (Suarez-Mendez et al., 2014).

Proteome allocations and respective metabolic phenotypes were then compared to the steady-state at the same (average) growth rate. First, we studied the maximum, minimum, and average enzyme saturation (V/V_max_) under dynamic conditions compared to the enzyme saturation under chemostat conditions (Table 3). Under dynamic conditions, the maximal enzyme saturation is much higher (up to 92% for the respiration reaction) than that under chemostat conditions (77% for respiration). Nevertheless, the average enzyme saturation over the whole cycle is actually lower than that under the reference chemostat state (for respiration, 25% compared to 77% at steady-state). This indicates that the proteome optimization to some extent focuses on the “peak” flux, especially for the large sectors of respiration and growth, indicating high usage of the available flux capacity while on average leaving a large overcapacity over the whole cycle. This enables a rapid consumption of the substrate as soon as it becomes available, which was the optimization criteria.

**TABLE 3:** Enzyme saturation, i.e., v/v_max_ under dynamic feeding conditions compared to steady-state (both at a dilution rate of D = 0.1 h-1). For dynamic conditions, v/v_max_ is calculated at the maximum rate during the cycle and the minimum as well as the average over the cycle. Upt, uptake; UGlc, upper glycolysis; LGlc, lower glycolysis; Ferm, fermentation; Esnk, electron sink/glycerol pathway; Resp, respiration; TrSn, trehalose synthesis; TrDg, trehalose degradation; Grwt, growth.

	Upt	Uglc	LGlc	Ferm	Esnk	Resp	TrSn	TrDg	Grwt
Max V/V_max_ ratio under FF	6%	19%	8%	25%	46%	92%	79%	11%	100%
Min V/V_max_ ratio under FF	<1%	<1%	<1%	<1%	<1%	1%	<1%	4%	<1%
Average V/V_max_ ratio under FF	1%	3%	1%	4%	7%	25%	10%	7%	24%
V/V_max_ ratio under chemostat	<1%	2%	6%	-	13%	77%	-	-	74%

**FIGURE 4:**
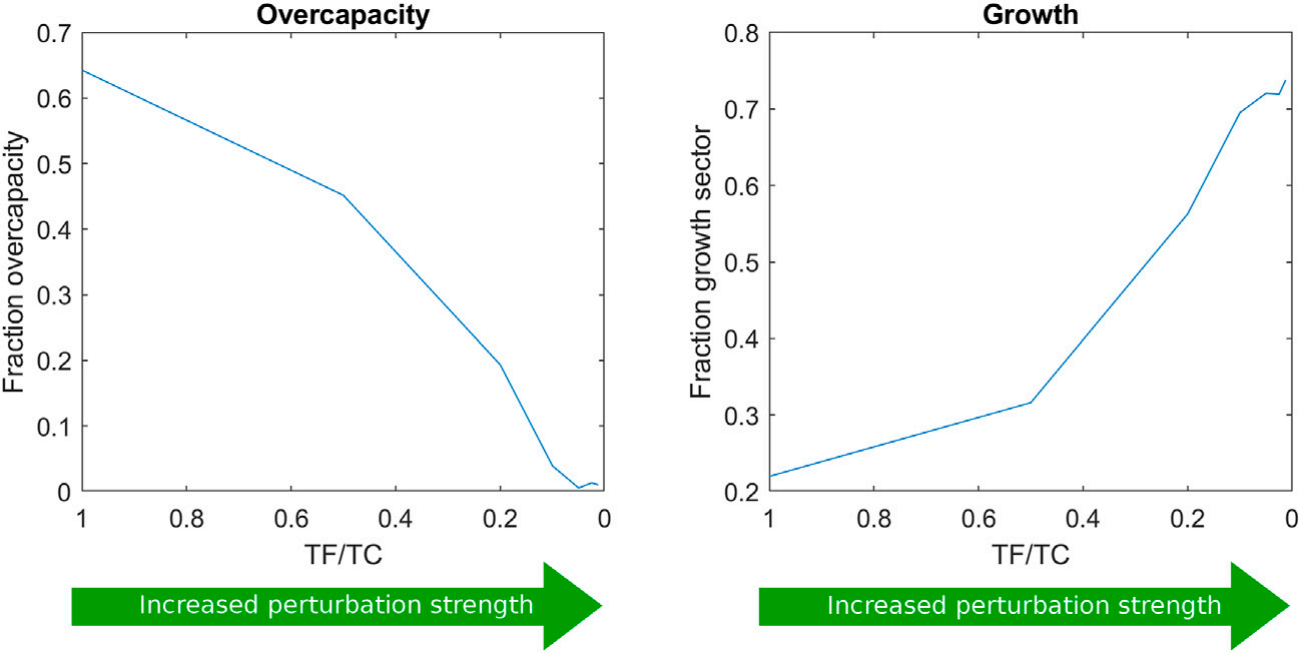
Proteome allocation as a function of the ratio of feeding time over cycle time (TF/TC). Further proteome sector fractions are shown in Supplementary Material S5.

We were now curious to see how the perturbation strength would influence the proteome allocation. Therefore, the length of the feeding period was varied at the same average dilution rate, resulting in different substrate perturbation intensities. The respective predicted proteome allocations were calculated and compared (Figure 4) for the different ratios of feeding time over cycle time (TF/TC). TF/TC values were chosen as log2 increments from the experimentally used TF/TC value of 1/20 (Suarez-Mendez et al., 2014).

The growth sector fraction increased with the perturbation intensity, suggesting that this strategy was the most effective measure to survive the higher substrate concentration variations (from faster feeding) and consequently high flux dynamics. The growth reaction seemed to act as an efficient and fast sink for substrate and ATP. However, in reality, the growth sector does not consist of a single reaction and may not be able to provide a rapid response upon glucose influx. For this reason, two other scenarios were additionally evaluated: 1) the regulation of the trehalose cycle upon repeated substrate pulses and 2) the regulation of the ratio between upper and lower glycolysis (see Supplementary Material S6).

### Impact of the Proteome Fraction on the Trehalose Cycle

**FIGURE 5:**
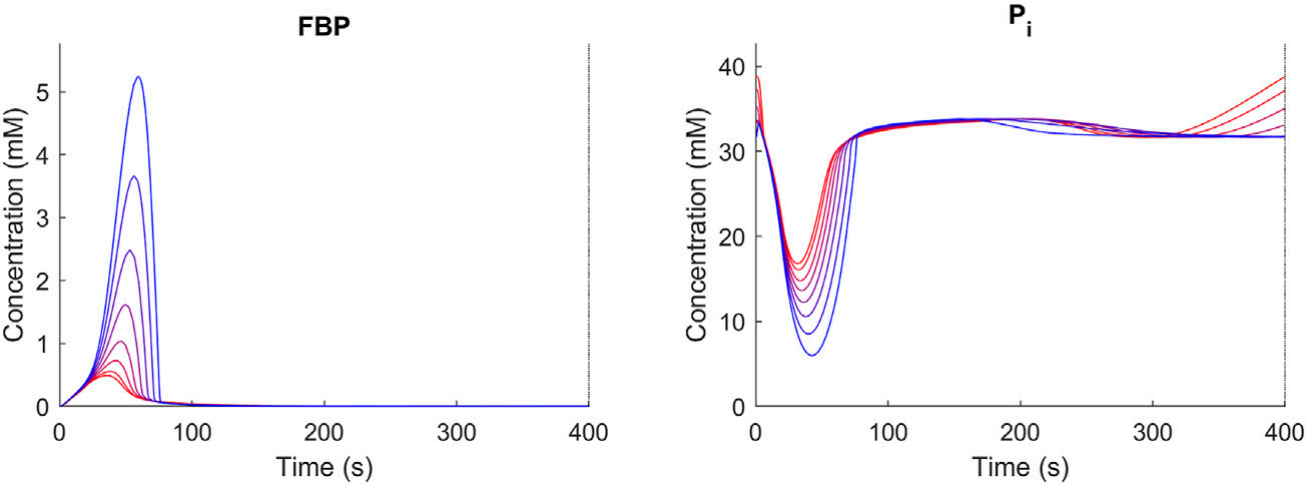
Concentration time course over repetitive cycles (D = 0.1 h^−1^, TF/TC = 0.05) for different trehalose sector fractions (blue = 0.1 red 1%). Shown are FBP and Pi as representative metabolites. For trehalose sector fractions <0.1%, no stable cycles were obtained.

The trehalose cycle has been described to function as a “safety valve” upon large changes in the glycolytic flux (Thevelein and Hohmann, 1995; Blomberg, 2000; van Heerden et al., 2014; Vicente et al., 2018). Under dynamic conditions in yeast, it was found that a significant amount of imported glucose was recycled through the trehalose cycle, especially during periods of high flux changes (Suarez-Mendez et al., 2017). To evaluate the effect of storage metabolism activity under dynamic conditions, the reference condition [D = 0.1 h-1, TF/TC = 0.05, (Suarez-Mendez et al., 2014)], was further analyzed. We varied the trehalose sector size between 0 and 1% (Figure 5) and compared the response of metabolism using FBP and Pi as indicators. A balanced metabolic response will lead to repetitive cycles in FBP and Pi. Such repetitive response was observed for proteomes with a trehalose sector larger than 0.1%. Increasing the trehalose sector above 0.1% leads to reduced fluctuations in G6P/FBP and Pi, suggesting a more robust metabolic response. Simulated changes in FBP and Pi are in line with results from previous work by van Heerden et al. (2014).

### Comparison of the Model Predictions to Experimental Proteomes

**FIGURE 6:**
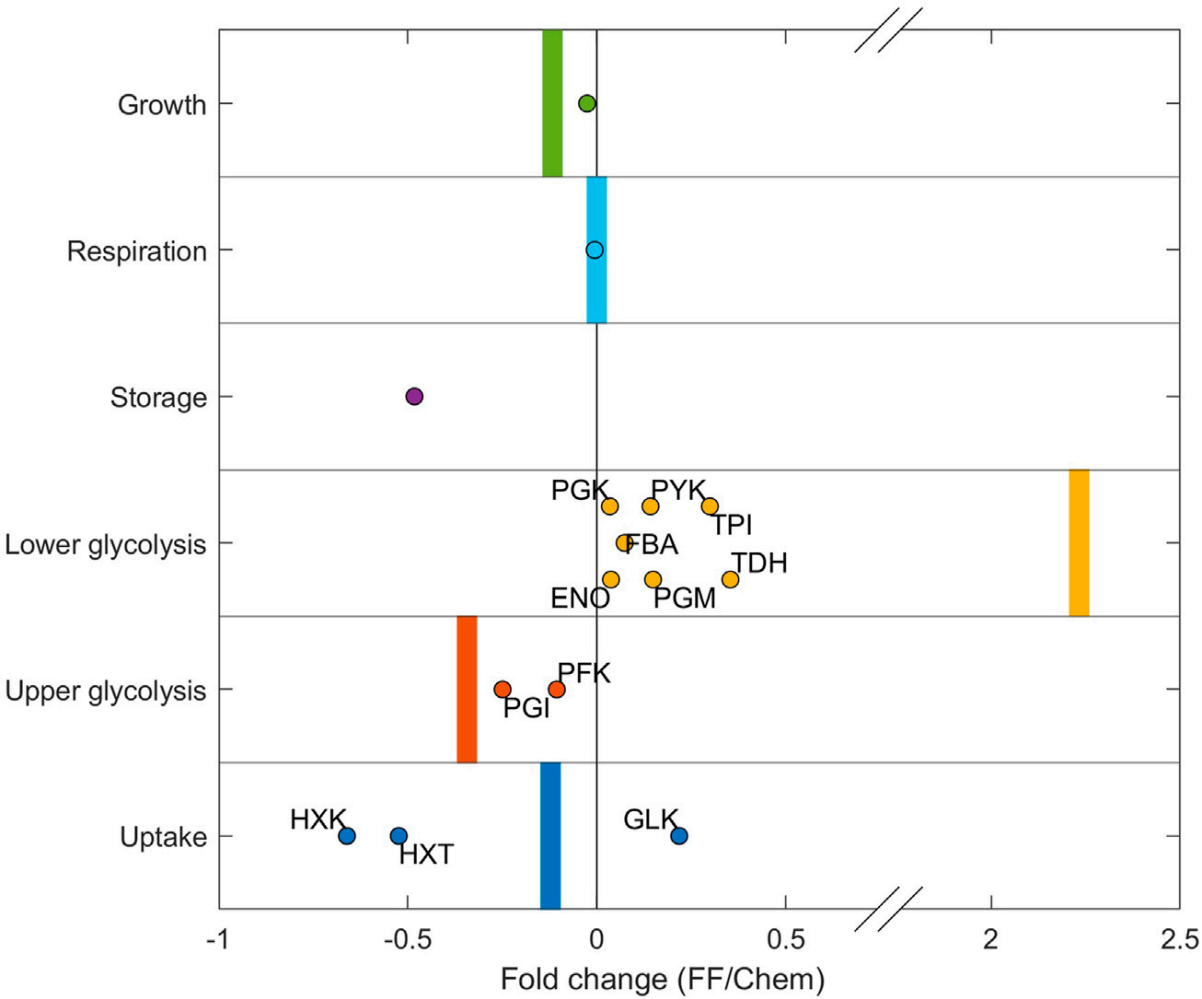
Protein concentration fold change from chemostat to feast/famine cultivation. The experimental fold change individual proteins are displayed as dots. Proteins of trehalose/glycogen storage, ribosomes, and oxidative phosphorylation were used as proxies for the storage, growth, and respiration sectors, respectively. Simulation fold changes for each sector are shown as vertical bars. The simulated storage sector for steady-state conditions was 0 and increased under simulated feast/famine conditions. As such, no fold change could be calculated, and therefore this fold change is not shown.

To evaluate the prediction accuracy and trends of the predicted proteomes under dynamic conditions, the simulated proteome adaptation from chemostat to feast/famine conditions was compared with the experimentally measured proteome fold changes between chemostat and feast/famine conditions (Verhagen et al., 2022) (Figure 6). Proteins of trehalose/glycogen storage, ribosomes, and oxidative phosphorylation were used as proxies for the storage, growth, and respiration sectors, respectively (proteins categorized in the same way as calibration approach, see Methods).

The model predicted the experimentally observed changes in upper and lower glycolysis (Figure 6). The enzyme TDH catalyzes the glyceraldehyde dehydrogenase reaction (TDH), which forms ATP using Pi. However, if upper and lower glycolysis reactions are imbalanced during high fluxes, this reaction becomes a bottleneck, leading to the accumulation of FBP and subsequently to an imbalanced metabolism. Therefore, it was expected that TDH had to be upregulated under substrate-fluctuating conditions to facilitate balanced intermediates, which was reflected in both the model predictions as well as in the experimental dataset. The predicted change in lower glycolysis is larger than that in the experimental data. This is likely caused by the fact that simulated proteomes for chemostat conditions contain no overcapacity in the lower glycolysis sector, whereas experimental proteomes under chemostat conditions appear to contain more overcapacity in this proteome sector. As such, the fold change between measured and simulated values is higher. Furthermore, the model reproduced the average change observed for the uptake sector, although it should be noted that effects of individual iso-enzymes (especially with regard to HXK/GLK, which catalyzes the first step of glycolysis) were not taken into account in the current model.

Significant deviations between experimental and predicted fractions were observed for the storage sector. This was significantly decreased experimentally, while the resource-dependent kinetic model predicted an increase. Experimentally, a decrease of 28%, from 0.25 to 0.2% of the proteome, was observed, while an increase to 0.2% of the proteome was predicted in the model. Possible reasons for this difference in sector size could be: 1) the synthesis of trehalose has additional functions in the cell which are not represented in the model—it is described that trehalose plays an important role in different stress responses, including severe substrate limitation at low dilution rates (see also Supplementary Figure S3). 2) The measured and predicted proteomes do not include neither post-translational modifications, which are known to significantly affect the k_cat_’s of enzymes in the trehalose cycle (Sengupta et al., 2011), nor changes that could occur during cell-cycle progression.

Furthermore, there could also be a bias from the experimental setup–the differences in the trehalose sector, combined with the observed increase of the lower glycolysis sector, suggest that the experimental chemostat proteome is potentially already primed for dynamic environments, compared to experimental conditions, and as such is more robust than the predicted optimized chemostat proteomes.

## Conclusion and Outlook

In this work, we developed a proteome-dependent kinetic modeling framework that predicts the optimal proteome composition for defined extracellular dynamic conditions. The approach could reproduce observed complex metabolic phenomena, such as the Crabtree effect, including long-term adjustments under chemostat conditions.

Analysis of the predicted proteomes showed that under substrate-limiting conditions (i.e., low dilution rates) with close to constant extracellular concentrations, a significant part of the optimized proteome is not required (thus a lot of overcapacity). With increasing substrate availability and/or concentration fluctuations, this overcapacity is shown to decrease. Cells optimized for steady-state conditions were not able to survive these substrate perturbations. This suggests that in reality, when conditions are never as ideal and “optimal” as presented in the model simulations, cells already possess proteome adjustments to create a more robust metabolism, allowing them to cope effectively with external perturbations such as substrate gradients.

Such adjustments to perturbations were found when comparing steady-state and feast/famine condition predictions. The approach generated a stable phenotype and the predicted changes in proteome allocation, i.e., downregulation of uptake and upper glycolysis sectors and upregulation of the lower glycolysis sector were also found experimentally. This complex and strongly kinetics-dependent prediction highlights the relevance of kinetic properties also for the regulation of protein expression. Nevertheless, to achieve this prediction, some constraints, which had to be derived from experimental observations, had to be included: the maximum mitochondrial fraction and the glucose repression on fermentation. These boundaries seemed to be only stretched after very long-term evolution, as observed by Barford and Hall (1979). Following this observation, the model was used to predict the proteome composition and metabolic behavior of cells at different stages of adaptation, able to simulate differences in cultivation history. Thus, the modeling approach was able to cover a large range of conditions and evolution outcomes, which could be specifically relevant for the prediction of production process regimes running over a long time span.

## Data Availability

The dataset and the MATLAB scripts for the model presented in this study can be found in online repositories at: https://doi.org/10.4121/19008833, https://doi.org/10.4121/19074791.

## References

[B1] Abulesz E.-M. Lyberatos G. (1989). Periodic Operation of a Continuous Culture of Baker's Yeast. Biotechnol. Bioeng. 34, 741–749. 10.1002/bit.260340603 18588160

[B2] Barford J. P. Hall R. J. (1979). An Examination of the Crabtree Effect in *Saccharomyces cerevisiae*: the Role of Respiratory Adaptation. J. Gen. Microbiol. 114, 267–275. 10.1099/00221287-114-2-267

[B3] Basan M. Hui S. Okano H. Zhang Z. Shen Y. Williamson J. R. (2015). Overflow Metabolism in *Escherichia coli* Results from Efficient Proteome Allocation. Nature 528, 99–104. 10.1038/nature15765 26632588 PMC4843128

[B4] Basan M. (2018). Resource Allocation and Metabolism: the Search for Governing Principles. Curr. Opin. Microbiol. 45, 77–83. 10.1016/j.mib.2018.02.008 29544124

[B5] Blomberg A. (2000). Metabolic Surprises inSaccharomyces Cerevisiaeduring Adaptation to saline Conditions: Questions, Some Answers and a Model. FEMS Microbiol. Lett. 182, 1–8. 10.1111/j.1574-6968.2000.tb08864.x 10612722

[B6] Boer V. M. Crutchfield C. A. Bradley P. H. Botstein D. Rabinowitz J. D. (2010). Growth-limiting Intracellular Metabolites in Yeast Growing under Diverse Nutrient Limitations. MBoC 21, 198–211. 10.1091/mbc.e09-07-0597 19889834 PMC2801714

[B7] Brejning J. Jespersen L. (2002). Protein Expression during Lag Phase and Growth Initiation in *Saccharomyces cerevisiae* . Int. J. Food Microbiol. 75, 27–38. 10.1016/S0168-1605(01)00726-7 11999115

[B8] Chu D. Barnes D. J. (2016). The Lag-phase during Diauxic Growth Is a Trade-Off between Fast Adaptation and High Growth Rate. Sci. Rep. 6, 25191. 10.1038/srep25191 27125900 PMC4850433

[B9] Chubukov V. Gerosa L. Kochanowski K. Sauer U. (2014). Coordination of Microbial Metabolism. Nat. Rev. Microbiol. 12, 327–340. 10.1038/nrmicro3238 24658329

[B10] Cohen A. A. Geva-Zatorsky N. Eden E. Frenkel-Morgenstern M. Issaeva I. Sigal A. (2008). Dynamic Proteomics of Individual Cancer Cells in Response to a Drug. Science 322, 1511–1516. 10.1126/science.1160165 19023046

[B11] de Godoy L. M. F. Olsen J. V. Cox J. Nielsen M. L. Hubner N. C. Fröhlich F. (2008). Comprehensive Mass-Spectrometry-Based Proteome Quantification of Haploid versus Diploid Yeast. Nature 455, 1251–1254. 10.1038/nature07341 18820680

[B12] Dekel E. Alon U. (2005). Optimality and Evolutionary Tuning of the Expression Level of a Protein. Nature 436, 588–592. 10.1038/nature03842 16049495

[B13] Della-Bianca B. E. de Hulster E. Pronk J. T. van Maris A. J. A. Gombert A. K. (2014). Physiology of the Fuel Ethanol strainSaccharomyces cerevisiaePE-2 at Low pH Indicates a Context-dependent Performance Relevant for Industrial Applications. FEMS Yeast Res. 14, 1196–1205. 10.1111/1567-1364.12217 25263709

[B14] Elsemman I. E. Rodriguez Prado A. Grigaitis P. Garcia Albornoz M. Harman V. Holman S. W. (2022). Whole-cell Modeling in Yeast Predicts Compartment-specific Proteome Constraints that Drive Metabolic Strategies. Nat. Commun. 13, 801. 10.1038/s41467-022-28467-6 35145105 PMC8831649

[B15] Ertugay N. Hamamci H. (1997). Continuous Cultivation of Bakers' Yeast: Change in Cell Composition at Different Dilution Rates and Effect of Heat Stress on Trehalose Level. Folia Microbiol. 42, 463–467. 10.1007/BF02826554 9438349

[B16] Gerosa L. Sauer U. (2011). Regulation and Control of Metabolic Fluxes in Microbes. Curr. Opin. Biotechnol. 22, 566–575. 10.1016/j.copbio.2011.04.016 21600757

[B17] Goelzer A. Muntel J. Chubukov V. Jules M. Prestel E. Nölker R. (2015). Quantitative Prediction of Genome-wide Resource Allocation in Bacteria. Metab. Eng. 32, 232–243. 10.1016/j.ymben.2015.10.003 26498510

[B18] Goffeau A. Barrell B. G. Bussey H. Davis R. W. Dujon B. Feldmann H. (1996). Life with 6000 Genes. Science 274, 546–567. 10.1126/science.274.5287.546 8849441

[B19] Heyland J. Fu J. Blank L. M. (2009). Correlation between TCA Cycle Flux and Glucose Uptake Rate during Respiro-Fermentative Growth of *Saccharomyces cerevisiae* . Microbiology 155, 3827–3837. 10.1099/mic.0.030213-0 19684065

[B20] Jannasch H. W. (1967). Enrichments of Aquatic Bacteria in Continuous Culture. Archiv. Mikrobiol. 59, 165–173. 10.1007/BF00406328 4880240

[B21] Jõers A. Tenson T. (2016). Growth Resumption from Stationary Phase Reveals Memory in *Escherichia coli* Cultures. Sci. Rep. 6, 24055. 10.1038/srep24055 27048851 PMC4822139

[B22] Kanehisa M. Sato Y. Kawashima M. Furumichi M. Tanabe M. (2016). KEGG as a Reference Resource for Gene and Protein Annotation. Nucleic Acids Res. 44, D457–D462. 10.1093/nar/gkv1070 26476454 PMC4702792

[B23] Karim M. R. Kawanago H. Kadowaki M. (2014). A Quick Signal of Starvation Induced Autophagy: Transcription versus post-translational Modification of LC3. Anal. Biochem. 465, 28–34. 10.1016/j.ab.2014.07.007 25062852

[B24] Liebermeister W. Noor E. Flamholz A. Davidi D. Bernhardt J. Milo R. (2014). Visual Account of Protein Investment in Cellular Functions. Proc. Natl. Acad. Sci. U.S.A. 111, 8488–8493. 10.1073/pnas.1314810111 24889604 PMC4060655

[B25] Litsios A. Ortega Á. D. Wit E. C. Heinemann M. (2018). Metabolic-flux Dependent Regulation of Microbial Physiology. Curr. Opin. Microbiol. 42, 71–78. 10.1016/j.mib.2017.10.029 29154077

[B26] Mahadevan R. Edwards J. S. Doyle F. J. (2002). Dynamic Flux Balance Analysis of Diauxic Growth in *Escherichia coli* . Biophysical J. 83, 1331–1340. 10.1016/S0006-3495(02)73903-9 PMC1302231 12202358

[B27] Molenaar D. van Berlo R. de Ridder D. Teusink B. (2009). Shifts in Growth Strategies Reflect Tradeoffs in Cellular Economics. Mol. Syst. Biol. 5, 323. 10.1038/msb.2009.82 19888218 PMC2795476

[B28] Mori M. Marinari E. De Martino A. (2019). A Yield-Cost Tradeoff Governs *Escherichia coli*'s Decision between Fermentation and Respiration in Carbon-Limited Growth. Npj Syst. Biol. Appl. 5, 16. 10.1038/s41540-019-0093-4 31069113 PMC6494807

[B29] Nagaraj N. Alexander Kulak N. Cox J. Neuhauser N. Mayr K. Hoerning O. (2012). System-wide Perturbation Analysis with Nearly Complete Coverage of the Yeast Proteome by Single-Shot Ultra HPLC Runs on a Bench Top Orbitrap. Mol. Cell Proteomics 11, M111.013722. 10.1074/mcp.M111.013722 PMC3316726 22021278

[B30] Nilsson A. Nielsen J. (2016). Metabolic Trade-Offs in Yeast Are Caused by F1F0-ATP Synthase. Sci. Rep. 6, 22264. 10.1038/srep22264 26928598 PMC4772093

[B31] Orth J. D. Thiele I. Palsson B. Ø. (2010). What Is Flux Balance Analysis? Nat. Biotechnol. 28, 245–248. 10.1038/nbt.1614 20212490 PMC3108565

[B32] Pincus D. Resnekov O. Reynolds K. A. (2017). An Evolution-Based Strategy for Engineering Allosteric Regulation. Phys. Biol. 14, 025002. 10.1088/1478-3975/aa64a4 28266924 PMC5943710

[B33] Postma E. Verduyn C. Scheffers W. A. Van Dijken J. P. (1989). Enzymic Analysis of the crabtree Effect in Glucose-Limited Chemostat Cultures of *Saccharomyces cerevisiae* . Appl. Environ. Microbiol. 55, 468–477. 10.1128/aem.55.2.468-477.1989 2566299 PMC184133

[B34] Reimers A.-M. Knoop H. Bockmayr A. Steuer R. (2017). Cellular Trade-Offs and Optimal Resource Allocation during Cyanobacterial Diurnal Growth. Proc. Natl. Acad. Sci. U.S.A. 114, E6457–E6465. 10.1073/pnas.1617508114 28720699 PMC5547584

[B35] Rieger M. KAPpeli O. Fiechter A. (1983). The Role of Limited Respiration in the Incomplete Oxidation of Glucose by Saccharomyces Cerevisiae. Microbiology 129, 653–661. 10.1099/00221287-129-3-653

[B36] Rügen M. Bockmayr A. Steuer R. (2015). Elucidating Temporal Resource Allocation and Diurnal Dynamics in Phototrophic Metabolism Using Conditional FBA. Sci. Rep. 5, 15247. 10.1038/srep15247 26496972 PMC4620596

[B37] Schuetz R. Kuepfer L. Sauer U. (2007). Systematic Evaluation of Objective Functions for Predicting Intracellular Fluxes in *Escherichia coli* . Mol. Syst. Biol. 3, 119. 10.1038/msb4100162 17625511 PMC1949037

[B38] Sengupta S. Chaudhuri P. Lahiri S. Dutta T. Banerjee S. Majhi R. (2011). Possible Regulation of Trehalose Metabolism by Methylation in *Saccharomyces cerevisiae* . J. Cel. Physiol. 226, 158–164. 10.1002/jcp.22317 20648561

[B39] Smallbone K. Malys N. Messiha H. L. Wishart J. A. Simeonidis E. (2011). “Building a Kinetic Model of Trehalose Biosynthesis in *Saccharomyces cerevisiae* ,” in Methods in Enzymology. 1st ed (Elsevier), 355–370. 10.1016/B978-0-12-385118-5.00018-9 21943906

[B40] Suarez-Mendez C. Sousa A. Heijnen J. Wahl A. (2014). Fast “Feast/Famine” Cycles for Studying Microbial Physiology under Dynamic Conditions: A Case Study with *Saccharomyces cerevisiae* . Metabolites 4, 347–372. 10.3390/metabo4020347 24957030 PMC4101510

[B41] Suarez-Mendez C. A. Hanemaaijer M. ten Pierick A. Wolters J. C. Heijnen J. J. Wahl S. A. (2016). Interaction of Storage Carbohydrates and Other Cyclic Fluxes with central Metabolism: A Quantitative Approach by Non-stationary 13 C Metabolic Flux Analysis. Metab. Eng. Commun. 3, 52–63. 10.1016/j.meteno.2016.01.001 29468113 PMC5779734

[B42] Suarez-Mendez C. A. Ras C. Wahl S. A. (2017). Metabolic Adjustment upon Repetitive Substrate Perturbations Using Dynamic 13C-Tracing in Yeast. Microb. Cel Fact 16, 161. 10.1186/s12934-017-0778-6 PMC5613340 28946905

[B43] Suarez-Mendez C. A. (2015). Dynamics of Storage Carbohydrates Metabolism in *Saccharomyces cerevisiae* . Delft, Netherlands: Delft University of Technology. 10.4233/UUID:2504BD76-9811-4D3C-A66B-3AAE7FBB40B5

[B44] Teusink B. Passarge J. Reijenga C. A. Esgalhado E. Van Der Weijden C. C. Schepper M. (2000). Can Yeast Glycolysis Be Understood in Terms of *In Vitro* Kinetics of the Constituent Enzymes? Testing Biochemistry. Eur. J. Biochem. 267, 5313–5329. 10.1046/j.1432-1327.2000.01527.x 10951190

[B45] Thevelein J. M. Hohmann S. (1995). Trehalose Synthase: Guard to the Gate of Glycolysis in Yeast? Trends Biochem. Sci. 20, 3–10. 10.1016/S0968-0004(00)88938-0 7878741

[B46] van Heerden J. H. Wortel M. T. Bruggeman F. J. Heijnen J. J. Bollen Y. J. M. Planqué R. (2014). Lost in Transition: Start-Up of Glycolysis Yields Subpopulations of Nongrowing Cells. Science 343, 1245114. 10.1126/science.1245114 24436182

[B47] Van Hoek P. Van Dijken J. P. Pronk J. T. (1998). Effect of Specific Growth Rate on Fermentative Capacity of Baker's Yeast. Appl. Environ. Microbiol. 64, 4226–4233. 10.1128/AEM.64.11.4226-4233.1998 9797269 PMC106631

[B48] Varma A. Palsson B. O. (1994). Stoichiometric Flux Balance Models Quantitatively Predict Growth and Metabolic By-Product Secretion in Wild-type *Escherichia coli* W3110. Appl. Environ. Microbiol. 60, 3724–3731. 10.1128/aem.60.10.3724-3731.1994 7986045 PMC201879

[B49] Vazquez A. Beg Q. K. deMenezes M. A. Ernst J. Bar-Joseph Z. Barabási A.-L. (2008). Impact of the Solvent Capacity Constraint on *E. coli* Metabolism. BMC Syst. Biol. 2, 7. 10.1186/1752-0509-2-7 18215292 PMC2270259

[B50] Verhagen K. J. A. Eerden S. A. Wahl S. A. (2022). Data from: Dataset Proteomics: Analysis of Change in Protein Expression in *Saccharomyces cerevisiae* upon Shift from Glucose Chemostat to Feast/famine Regime. 4TU.ResearchData. 10.4121/19008833

[B51] Vicente R. L. Spina L. Gómez J. P. L. Dejean S. Parrou J.-L. François J. M. (2018). Trehalose-6-phosphate Promotes Fermentation and Glucose Repression in *Saccharomyces cerevisiae* . Microb. Cel 5, 444–459. 10.15698/mic2018.10.651 PMC6206404 30386789

[B52] Vos T. Hakkaart X. D. V. de Hulster E. A. F. van Maris A. J. A. Pronk J. T. Daran-Lapujade P. (2016). Maintenance-energy Requirements and Robustness of *Saccharomyces cerevisiae* at Aerobic Near-Zero Specific Growth Rates. Microb. Cel Fact 15, 1–20. 10.1186/s12934-016-0501-z PMC4912818 27317316

[B53] Wegner A. Meiser J. Weindl D. Hiller K. (2015). How Metabolites Modulate Metabolic Flux. Curr. Opin. Biotechnol. 34, 16–22. 10.1016/j.copbio.2014.11.008 25461507

[B54] Wu L. van Dam J. Schipper D. Kresnowati M. T. A. P. Proell A. M. Ras C. (2006). Short-Term Metabolome Dynamics and Carbon, Electron, and ATP Balances in Chemostat-Grown *Saccharomyces cerevisiae* CEN.PK 113-7D Following a Glucose Pulse. Appl. Environ. Microbiol. 72, 3566–3577. 10.1128/AEM.72.5.3566-3577.2006 16672504 PMC1472385

[B55] Zhao Q. Stettner A. I. Reznik E. Paschalidis I. C. Segrè D. (2016). Mapping the Landscape of Metabolic Goals of a Cell. Genome Biol. 17, 109. 10.1186/s13059-016-0968-2 27215445 PMC4878026

