# Supplementary material for: Predicting Metabolic Adaptation Under Dynamic Substrate Conditions Using a Resource-Dependent Kinetic Model: A Case Study Using Saccharomyces cerevisiae
Source: Front Mol Biosci. 2022 May 16;9:863470. doi: 10.3389/fmolb.2022.863470 (PMC9149170; doi:10.3389/fmolb.2022.863470)
Supplement: Supplementary file 1 [file DataSheet1.docx]

Supplementary Material

#
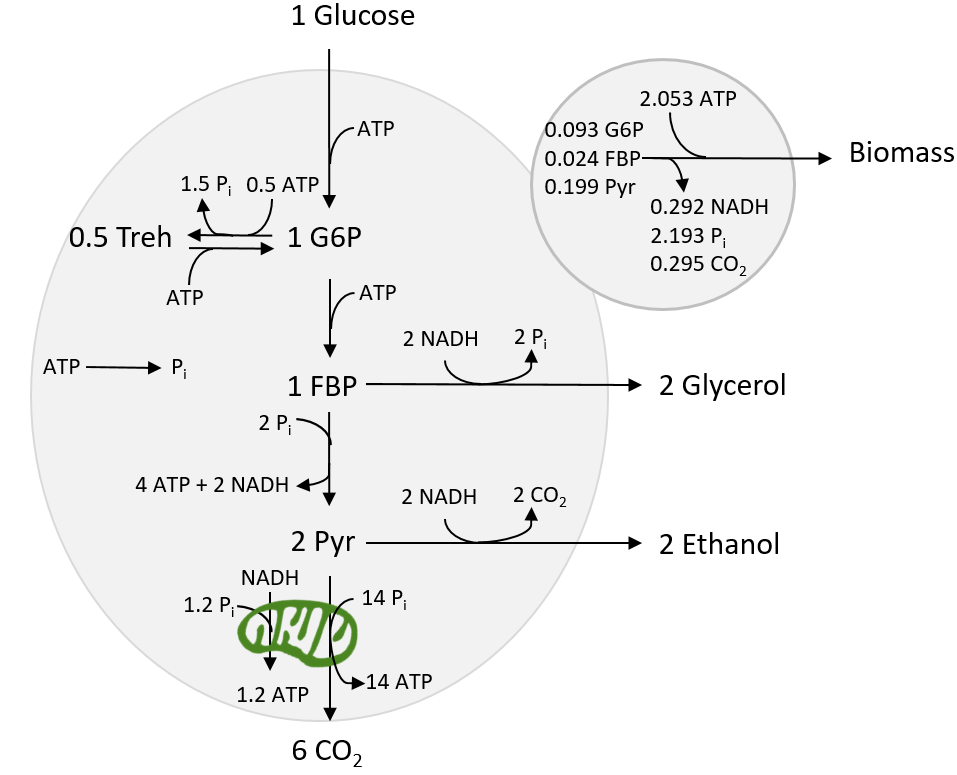
Model structure

Figure S1. Map of the metabolic network in this model

First, we tag glucose, ethanol, glycerol and biomass with the label EC, and the other compounds with the label IC, then the system of equations becomes:

$$\left\{ \begin{aligned} \frac{dc_{EC}}{dt}=D_{(t)}\cdot\left( c_{EC,in}-c_{EC} \right)+S_{EC} \cdot v\cdot c_{x} \\ \frac{dc_{IC}}{dt}=S_{IC}\cdot v\cdot\rho_{x} \end{aligned} \right.$$

In this model, 4 variables are user supplied:

- φ : an 9-dimensional proteome allocation vector (should add up to one)
- D_avg_ : the average dilution rate (for batch, set to 0)
- t_cycle_ : the length of a feast-famine cycle
- t_feed_ : the length of the feed phase of the feast-famine cycle (for chemostat, set equal to t_cycle_)

To compensate D for the feast/famine operation, D_(t)_ is defined as followed:

$$D_{(t)}=\left\{ \begin{aligned} D_{avg} \cdot\frac{t_{\mathrm{cycle}}}{t_{\mathrm{feed}}} in the feed phase \\ 0 outside feed phase \end{aligned} \right.$$

The concentrations of ADP and NAD are calculated as followed:

$$c_{ADP}=c_{AxP}-c_{ATP} with c_{AxP}$$

$$c_{NAD}=c_{NADx}-c_{NADH} with c_{NADx}$$

## Stoichiometry

Table S1. The stoichiometric matrix S of the metabolic model.

|  | Upt | UGlc | LGlc | Ferm | Esnk | TCA | NDE | TrSn | TrDg | Grwt | Mtn |
| --- | --- | --- | --- | --- | --- | --- | --- | --- | --- | --- | --- |
| Glucose | -1 | 0 | 0 | 0 | 0 | 0 | 0 | 0 | 0 | 0 | 0 |
| Biomass | 0 | 0 | 0 | 0 | 0 | 0 | 0 | 0 | 0 | 1 | 0 |
| Ethanol | 0 | 0 | 0 | 1 | 0 | 0 | 0 | 0 | 0 | 0 | 0 |
| Glycerol | 0 | 0 | 0 | 0 | 1 | 0 | 0 | 0 | 0 | 0 | 0 |
| ATP | -1 | -1 | 2 | 0 | 0 | 7/5 | 1.2 | -1 | -2 | -2.0531 | -1 |
| NADH | 0 | 0 | 1 | -1 | -1 | 0 | -1 | 0 | 0 | 0.2916 | 0 |
| P_i_ | 0 | 0 | -1 | 0 | 1 | -7/5 | -1.2 | 3 | 0 | 2.1930 | 1 |
| G6P | 1 | -1 | 0 | 0 | 0 | 0 | 0 | -2 | 2 | -0.0927 | 0 |
| FBP | 0 | 1 | -0.5 | 0 | -0.5 | 0 | 0 | 0 | 0 | -0.0236 | 0 |
| PYR | 0 | 0 | 1 | -1 | 0 | -1/5 | 0 | 0 | 0 | -0.1992 | 0 |
| Treh | 0 | 0 | 0 | 0 | 0 | 0 | 0 | 1 | -1 | 0 | 0 |
| (CO_2_)* | 0 | 0 | 0 | 1 | 0 | 3/5 | 0 | 0 | 0 | 0.2954 | 0 |

* CO_2_ is not simulated in the ODE system, but this stoichiometry was used for calculating q_CO2_

**Flux vector as used in ODE system:**

$$v= \left( \begin{matrix} v_{upt} \\ v_{uglc} \\ v_{lglc} \\ v_{ferm} \\ v_{esnk} \\ v_{TCA} \\ v_{NDE} \\ v_{trsn} \\ v_{trdg} \\ \mu\\ v_{mtn} \end{matrix} \right)$$

## Rate equations

### Uptake

$$v_{upt}=\varphi_{upt} \cdot k_{cat,upt}\cdot\frac{c_{Glc}}{K_{m,upt,Glc}+c_{Glc}} \cdot\frac{c_{ATP}}{K_{m,upt,ATP}+c_{ATP}}$$

The value of $\varphi_{upt}$ is capped to $\varphi_{upt}^{max}$, to reflect the limited space for proteins on the membrane. Any proteome allocated to this sector above this value is ignored.

### Upper glycolysis

$$v_{uglc}=\varphi_{uglc} \cdot k_{cat,uglc}\cdot\frac{c_{G6P}}{K_{m,uglc,G6P}+c_{G6P}} \cdot\frac{c_{ATP}}{K_{m,uglc,ATP}+c_{ATP}}\cdot\frac{K_{i,uglc,ATP}^{n_{uglc,ATP}}}{K_{i,uglc,ATP}^{n_{uglc,ATP}}+c_{ATP}^{n_{uglc,ATP}}}$$

### Lower glycolysis

$$v_{lglc}=\varphi_{lglc} \cdot k_{cat,lglc}\cdot\frac{c_{FBP}}{K_{m,lglc,FBP}+c_{FBP}} \cdot\frac{c_{ADP}}{K_{m,lglc,ADP}+c_{ADP}}\cdot\frac{c_{P_{i}}}{K_{m,lglc,P_{i}}+c_{P_{i}}}\cdot\frac{c_{NADH}}{c_{NADx}}$$

### Fermentation

$$v_{ferm}=\varphi_{ferm} \cdot k_{cat,ferm}\cdot\frac{c_{PYR}^{n_{ferm,PYR}}}{K_{m,ferm,PYR}^{n_{ferm,PYR}}+c_{PYR}^{n_{ferm,PYR}}} \cdot\frac{c_{NADH}^{n_{ferm,NADH}}}{K_{m,ferm,NADH}^{n_{ferm,NADH}}+c_{NADH}^{n_{ferm,NADH}}}$$

To regulate the activation of fermentation, a critical glucose concentration is set, equal to the residual glucose concentration at a dilution rate of 0.28 h^-1^ (0.057 mM).

### Glycerol electron sink

$$v_{esnk}=\varphi_{esnk} \cdot k_{cat,esnk}\cdot\frac{c_{FBP}}{K_{m,esnk,FBP}+c_{FBP}}\cdot\frac{c_{NADH}}{K_{m,esnk,NADH}+c_{NADH}}$$

### Respiration (pyruvate-dependent and NADH-dependent)

$$v_{TCA}=\varphi_{resp} \cdot k_{cat,resp}\cdot\frac{c_{PYR}}{K_{m,resp,PYR}+c_{PYR}}\cdot\frac{c_{ADP}^{n_{resp,ADP}}}{K_{m,resp,ADP}^{n_{resp,ADP}}+c_{ADP}^{n_{resp,ADP}}}\cdot\frac{c_{P_{i}}^{n_{resp,P_{i}}}}{K_{m,resp,P_{i}}^{n_{resp,P_{i}}}+c_{P_{i}}^{n_{resp,P_{i}}}}$$

$$v_{NDE}=\varphi_{resp} \cdot k_{cat,resp}\cdot\frac{c_{NADH}}{K_{m,resp,NADH}+c_{NADH}}\cdot\frac{c_{ADP}^{n_{resp,ADP}}}{K_{m,resp,ADP}^{n_{resp,ADP}}+c_{ADP}^{n_{resp,ADP}}}\cdot\frac{c_{P_{i}}^{n_{resp,P_{i}}}}{K_{m,resp,P_{i}}^{n_{resp,P_{i}}}+c_{P_{i}}^{n_{resp,P_{i}}}}$$

If $v_{TCA}+v_{NDE}>\varphi_{resp} \cdot k_{cat,resp}$ (if ETC capacity is exceeded)

Then $v_{NDE}= \varphi_{resp} \cdot k_{cat,resp}-v_{TCA}$ (TCA cycle gets priority, rest of $\varphi_{resp}$ is used by NDE (mitochondrial external NADH dehydrogenase)).

The value of $\varphi_{resp}$ is capped to $\varphi_{resp}^{max}$, to reflect the limited space for proteins on the membrane. Any proteome allocated to this sector above this value is ignored.

### Trehalose synthesis

$$v_{trsn}=\varphi_{treh} \cdot k_{cat,trsn}\cdot\frac{c_{G6P}^{n_{treh,G6P}}}{K_{m,trsn,G6P}^{n_{treh,G6P}}+c_{G6P}^{n_{treh,G6P}}}\cdot\frac{c_{ATP}}{K_{m,trsn,ATP}+c_{ATP}}$$

UTP of the original model (Smallbone et al., 2011) has been replaced by ATP assuming thermodynamic equilibrium between these pools, i.e. it is assumed that the phosphorylation ratio of the uridylate and adenylate pools is quasi-constant, and that the adenylate pool is about 3.5 times as big as the uridylate pool (Suarez-Mendez et al., 2016).

### Trehalose degradation

$$v_{trsn}=\varphi_{treh} \cdot k_{cat,trdg}\cdot\frac{c_{treh}}{K_{m,trdg,treh}+c_{treh}}\cdot\frac{c_{ATP}}{K_{m,trdg,ATP}+c_{ATP}}$$

### Growth

$$\mu=\varphi_{grwt} \cdot k_{cat,grwt}\cdot\frac{c_{ATP}^{n_{grwt,ATP}}}{K_{m,grwt,ATP}^{n_{grwt,ATP}}+c_{ATP}^{n_{grwt,ATP}}}\cdot\frac{c_{G6P}^{n_{grwt,G6P}}}{K_{m,grwt,G6P}^{n_{grwt,G6P}}+c_{G6P}^{n_{grwt,G6P}}}\cdot\frac{c_{FBP}^{n_{grwt,FBP}}}{K_{m,grwt,FBP}^{n_{grwt,FBP}}+c_{FBP}^{n_{grwt,FBP}}}\cdot\frac{c_{PYR}^{n_{grwt,PYR}}}{K_{m,grwt,PYR}^{n_{grwt,PYR}}+c_{PYR}^{n_{grwt,PYR}}} \cdot\frac{c_{NAD}^{n_{grwt,NAD}}}{K_{m,grwt,NAD}^{n_{grwt,NAD}}+c_{NAD}^{n_{grwt,NAD}}}$$

### Maintenance

$$v_{mtn}=k_{cat,mtn}\cdot\frac{c_{ATP}}{K_{m,mtn,ATP}+c_{ATP}}$$

## Used parameter values

Table S2. Estimated k_cat_ parameters without multiplication (k_cat,i_), multiplication factors and the final k_cat_ parameters estimated for each protein sector based on the proteome and fluxome of yeast in batch conditions (de Godoy et al., 2008; Heyland et al., 2009).

| Parameter | Value (mol∙Cmol_x_^-1^∙h^-1^) | Origin |
| --- | --- | --- |
| K_cat,upt_ | 567.03 | Fitted to proteome data of (de Godoy et al., 2008) and flux distribution of (Heyland et al., 2009) |
| K_cat,uglc_ | 223.07 | Fitted to proteome data of (de Godoy et al., 2008) and flux distribution of (Heyland et al., 2009) |
| K_cat,lglc_ | 127.84 | Fitted to proteome data of (de Godoy et al., 2008) and flux distribution of (Heyland et al., 2009) |
| K_cat,ferm_ | 229.1 | Fitted to proteome data of (de Godoy et al., 2008) and flux distribution of (Heyland et al., 2009) |
| K_cat,esnk_ | 30.721 | Fitted to proteome data of (de Godoy et al., 2008) and flux distribution of (Heyland et al., 2009) |
| K_cat,resp_ | 3.501 | Fitted to proteome data of (de Godoy et al., 2008) and flux distribution of (Heyland et al., 2009) |
| K_cat,trsn_ | 5.919 | Fitted to proteome data of (de Godoy et al., 2008) and flux distribution of (Suarez-Mendez et al., 2017) |
| K_cat,trdg_ | 7.812 | Fitted to proteome data of (de Godoy et al., 2008) and flux distribution of (Suarez-Mendez et al., 2017) |
| K_cat,grwt_ | 0.578 | Fitted to proteome data of (de Godoy et al., 2008) and flux distribution of (Heyland et al., 2009) |
| K_cat,mtn_ | 0.0155 | (Vos et al., 2016) |

Table S3. The values for the K_m_ as used in this model.

| Parameter | Value | Origin |
| --- | --- | --- |
| K_M,upt,S_ | 10.286 mol∙m^-3^ | Fitted to model of (Teusink et al., 2000), multiplied by 10 to set residual glucose to realistic value in Crabtree experiment |
| K_M,upt,ATP_ | 0.4186 mol∙m^-3^ | Fitted to model of (Teusink et al., 2000) |
| K_M,uglc,G6P_ | 1.9811 mol∙m^-3^ | Fitted to model of (Teusink et al., 2000) |
| K_M,uglc,ATP_ | 0.1564 mol∙m^-3^ | Fitted to model of (Teusink et al., 2000) |
| K_i,uglc,ATP_ | 1.68 mol∙m^-3^ | Fitted to model of (Teusink et al., 2000) |
| K_M,lglc,FBP_ | 0.736 mol∙m^-3^ | Fitted to model of (Teusink et al., 2000) |
| K_M,lglc,ADP_ | 0.27 mol∙m^-3^ | Fitted to model of (Teusink et al., 2000) |
| K_M,lglc,Pi_ | 4.78 mol∙m^-3^ | Fitted to model of (Teusink et al., 2000) |
| K_M,ferm,PYR_ | 0.866 mol∙m^-3^ | Fitted to model of (Teusink et al., 2000) |
| K_M,ferm,NADH_ | 0.01∙c_NADx_ | Arbitrarily chosen (much smaller than in vivo concentration) |
| K_M,esnk,FBP_ | 0.1648 mol∙m^-3^ | Fitted to model of (Teusink et al., 2000) |
| K_M,esnk,NADH_ | 0.0352 mol∙m^-3^ | Fitted to model of (Teusink et al., 2000) |
| K_M,mtn,ATP_ | 0.01∙c_AxP_ | Arbitrarily chosen (ATP cannot be used if it is depleted) |
| K_M,resp,PYR_ | 0.8 mol∙m^-3^ | K_m_ of isolated mitochondria for pyruvate (Pronk et al., 1996) |
| K_M,resp,NADH_ | 0.03 mol∙m^-3^ | NDI kinetics, (de Vries and Grivell, 1988), assuming that NDE has same K_M_ |
| K_M,resp,ADP_ | 0.01∙c_AxP_ | Arbitrarily chosen (much smaller than in vivo concentration) |
| K_M,resp,Pi_ | 0.05 mol∙m^-3^ | Arbitrarily chosen (much smaller than in vivo concentration) |
| K_M,trsn,G6P_ | 0.973 mol∙m^-3^ | Fitted to model of (Smallbone et al., 2011) |
| K_M,trsn,ATP_ | 0.255 mol∙m^-3^ | Fitted to model of (Smallbone et al., 2011) |
| K_M,trdg,Treh_ | 5 mol∙m^-3^ | Fitted to model of (Smallbone et al., 2011) |
| K_M,trdg,ATP_ | 0.4186 mol∙m^-3^ | Fitted to model of (Smallbone et al., 2011) |
| K_M,grwt,ATP_ | 0.75∙c_AxP_ | Arbitrarily chosen so that µ is sensitive in energy charges around 0.8 |
| K_M,grwt,G6P_ | 0.35 mol∙m^-3^ | Arbitrarily chosen (much smaller than in vivo concentration) |
| K_M,grwt,FBP_ | 0.02 mol∙m^-3^ | Arbitrarily chosen (much smaller than in vivo concentration) |
| K_M,grwt,PYR_ | 0.2 mol∙m^-3^ | Arbitrarily chosen (much smaller than in vivo concentration) |
| K_M,grwt,NAD_ | 0.01∙c_NADx_ | Arbitrarily chosen (much smaller than in vivo concentration) |

Table S4. Miscellaneous parameter values used in this model.

| Parameter | Value | Origin |
| --- | --- | --- |
| c_Glc,in_ | 41.67 mol∙m^-3^ (7.5 g/l) | (Suarez-Mendez et al., 2014) |
| c_x,in_ | 0 | Sterile medium |
| C_EtOH,in_ | 0 | No ethanol in medium |
| C_glyc,in_ | 0 | No glycerol in medium |
| ρ_x_ | 26656 mol_x_∙m^-3^ | Derived from (Illmer et al., 1999) and (Lamprecht et al., 1976)  assuming 1 mol biomass weighs 24.6 g_x_ |
| C_AxP_ | 8.28 mol∙m^-3^ | (Suarez-Mendez et al., 2014) |
| C_NADx_ | 1.46 mol∙m^-3^ | (Suarez-Mendez et al., 2014) |
| n_uglc,ATP_ | 2 | Fitted to model of (Teusink et al., 2000) |
| n_treh,G6P_ | 1.58 | Fitted to model of (Smallbone et al., 2011) |
| n_ferm,PYR_ | 1.9 | Fitted to model of (Teusink et al., 2000) |
| n_ferm,NADH_ | 25 | Arbitrarily chosen (large value for sharp sigmoid) |
| n_resp,ADP_ | 25 | Arbitrarily chosen (large value for sharp sigmoid) |
| n_resp,Pi_ | 25 | Arbitrarily chosen (large value for sharp sigmoid) |
| n_grwt,ATP_ | 25 | Arbitrarily chosen so that µ is sensitive in energy charges around 0.8 (assuming that the “safety valves” have not been triggered) |
| n_grwt,G6P_ | 10 | Arbitrarily chosen (large value for sharp sigmoid) |
| n_grwt,FBP_ | 10 | Arbitrarily chosen (large value for sharp sigmoid) |
| n_grwt,PYR_ | 10 | Arbitrarily chosen (large value for sharp sigmoid) |
| n_grwt,NAD_ | 25 | Arbitrarily chosen (large value for sharp sigmoid) |
| $\varphi_{upt}^{max}$ | 0.016 | Arbitrarily chosen, as twice the uptake sector in the proteome data from (de Godoy et al., 2008) |
| $\varphi_{resp}^{max}$ | 0.12 | Arbitrarily chosen to match the oxygen  consumption of (Van Hoek et al., 1998) |

## Objective function

Under steady-state conditions, the minimization of the residual substrate concentration was used as objective function. Under dynamic conditions, the minimization of a time-weighted average substrate concentration was used, to promote fast consumption of available substrate, therefore selecting for competitive proteomes:

$$\frac{\int_{0}^{t_{cycle}} c_{s}\cdot t dt}{\int_{0}^{t_{cycle}} tdt}$$

# Comparison of predicted flux distribution to experimental flux distribution

The k_cat_ parameters were calculated by estimating the fluxes that were obtained at half-saturation conditions with the experimental batch proteome (de Godoy et al., 2008). This is done with a proteome model in which all k_cat_ parameters equal one. Subsequently, the factor between these fluxes and the experimental flux distribution of (Heyland et al., 2009) is defined as the k_cat_ parameter for the corresponding reaction.

When the obtained parameters are used to simulate a chemostat experiment at a dilution rate of 0.4 h^-1^ the cells reach an unstable state because the flux capacity is not sufficient. This was assumed to be a result of both the conditions under which the parameters were estimated and the possibility of reserve flux capacity in the actual yeast proteome. The concentrations used for the estimation might be too low, as it is likely that the cells operate closer to saturation conditions when growing at the maximum growth rate. To correct the flux capacity and to enable accurate reproduction of the flux distribution of (Heyland et al., 2009), the estimated k_cat_ parameters are increased with specific factors (Supplementary Material 1), except for the k_cat_ for maintenance. These factors are found with a multi-start optimization aimed at minimizing the difference between the simulated fluxes and the flux distribution of (Heyland et al., 2009). The result of simulating a chemostat experiment at a dilution rate of 0.4 h^-1^ with the new k_cat_ parameters shows that the model can now successfully simulate an experiment with the experimental proteome allocation.

# Comparison of steady state model with experimental data at different dilution rates

Figure S2. Comparison of simulated steady-state fluxes at different dilution rates (orange line) with experimental data from (Suarez-Mendez et al., 2016) (black points). Upt = Uptake, UGlc = Upper Glycolysis, LGlc = Lower Glycolysis, Ferm = Fermentation, Esnk = Electron sink/glycerol pathway, Resp = Respiration

Figure S3. Comparison of simulated steady-state concentrations at different dilution rates (orange line) with experimental data from (Suarez-Mendez et al., 2016) (black points).

#
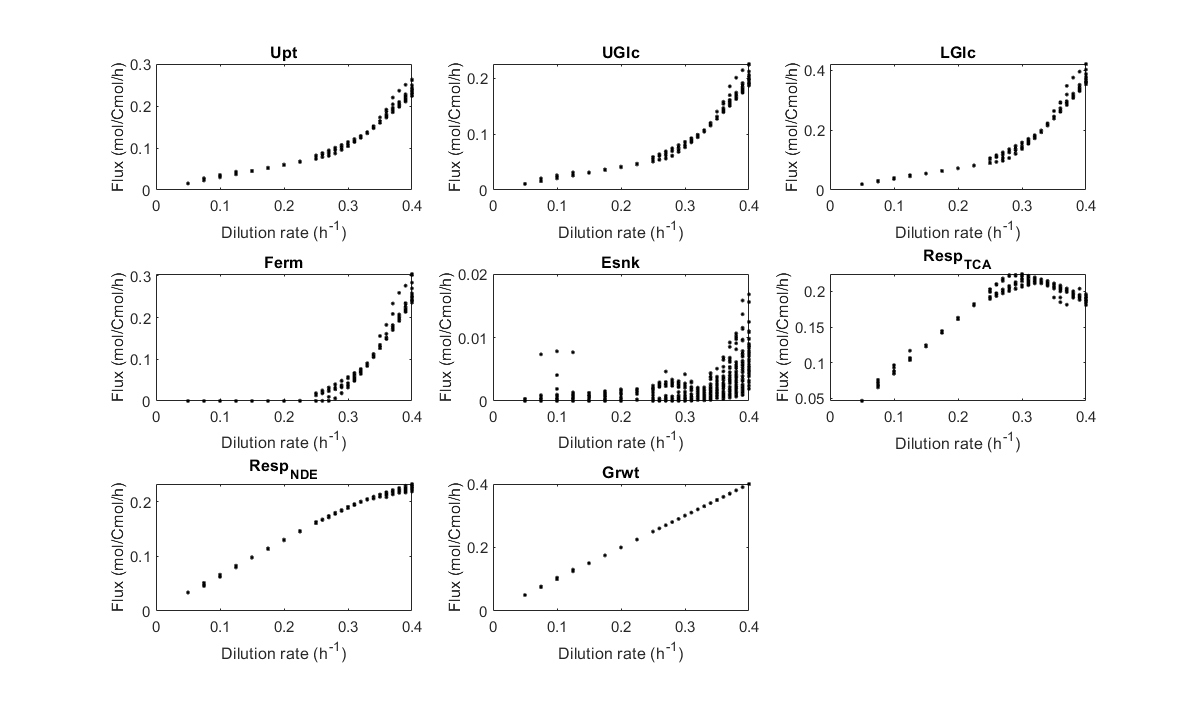
Sensitivity analysis of steady state model at different dilution rates

Figure S4. Sensitivity analysis of fluxes through each sector at different steady-state dilution rates. Per dilution rate, 40 simulations were evaluated. Especially around the critical dilution rate of 0.28 h^-1^, when the Crabtree effect occurs, variability with respect to respiration and fermentation is present, but overall limited variability is present in the fluxes of optimized proteomes.


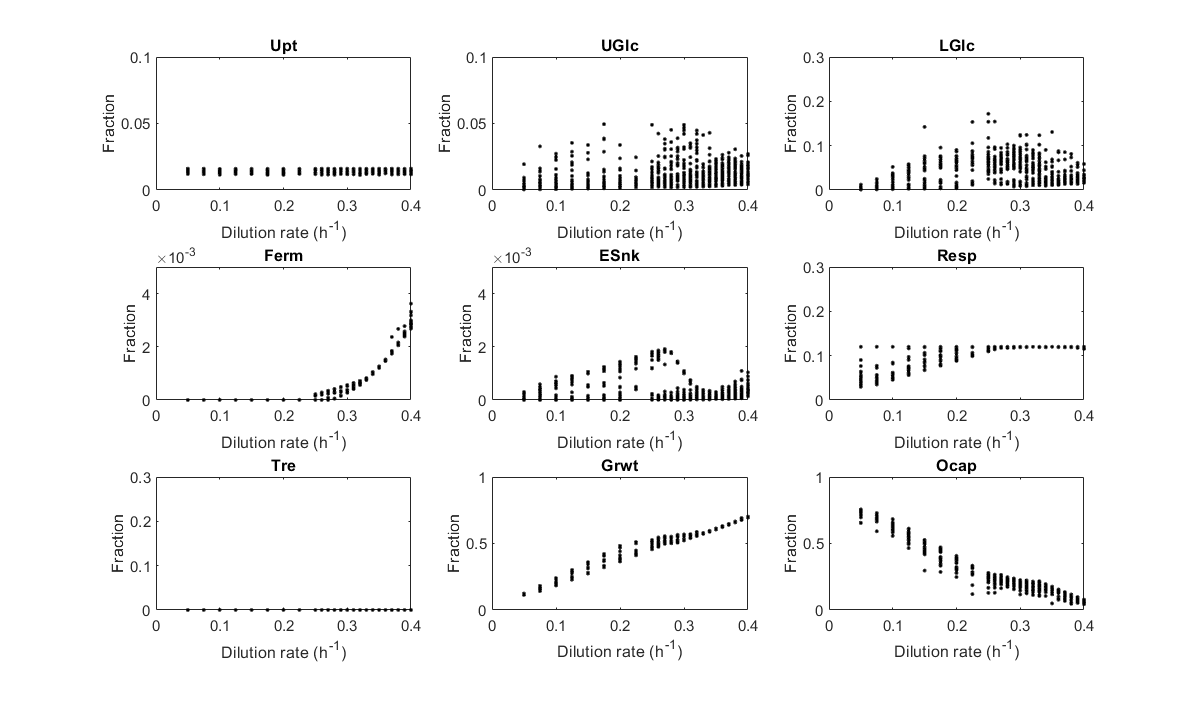
Figure S5. Sensitivity analysis of proteome composition at different steady-state dilution rates. Per dilution rate, 40 simulations were evaluated. High variability is especially present in the lower glycolysis and respiration sectors, Significantly affecting the amount of overcapacity present. This high variability does however not appear to have a large impact on the fluxes (Figure S4).

# Optimized proteome sector sizes at increasing feast/famine perturbation strength

Table S5. The optimized proteome sector sizes at increasing perturbation strength. The perturbation strength is expressed as the feeding time over the cycle time (TF/TC); a TF/TC of 1 is equal to chemostat, 0.05 is equal to an experimental pulse perturbation strength of a feast/famine cycle (Suarez-Mendez et al., 2014). Upt = Uptake, UGlc = Upper Glycolysis, LGlc = Lower Glycolysis, Ferm = Fermentation, Esnk = Electron sink/glycerol pathway, Resp = Respiration, TrSn = Trehalose synthesis, TrDg = Trehalose degradation, Grwt = Growth, Strc = Structural sector, Ocap = Overcapacity

| TF/TC | Upt | UGlc | LGlc | ESnk | Ferm | Resp | Tre | Growth | Strc | Ocap |
| --- | --- | --- | --- | --- | --- | --- | --- | --- | --- | --- |
| 1 | 0.0139 | 0.0035 | 0.004 | 0 | 0.0002 | 0.0535 | 0 | 0.2199 | 0.0625 | 0.6425 |
| 0.5 | 0.0154 | 0.0063 | 0.023 | 0.0005 | 0.0002 | 0.1141 | 0.002 | 0.3162 | 0.0708 | 0.4516 |
| 0.2 | 0.016 | 0.0023 | 0.0384 | 0.0002 | 0.0006 | 0.12 | 0.0045 | 0.5627 | 0.0618 | 0.1934 |
| 0.1 | 0.016 | 0.0061 | 0.0573 | 0.0031 | 0.0005 | 0.12 | 0.001 | 0.6951 | 0.0618 | 0.0391 |
| 0.05 | 0.016 | 0.0091 | 0.0585 | 0.0089 | 0.001 | 0.12 | 0.0013 | 0.7205 | 0.0592 | 0.0055 |
| 0.025 | 0.0136 | 0.0092 | 0.0471 | 0.0143 | 0.0006 | 0.12 | 0.0008 | 0.7193 | 0.0618 | 0.0134 |
| 0.0125 | 0.0134 | 0.009 | 0.0395 | 0.0063 | 0.0007 | 0.1197 | 0.0014 | 0.7379 | 0.0618 | 0.0102 |

# The ratio of upper over lower glycolysis and its effect on the stability of the glycolytic pathway

The phosphate ‘deadlock’, described by (van Heerden et al., 2014) to be dependent on the intracellular concentrations of Pi and FBP, is hypothesized (next to the trehalose cycle) to be additionally regulated by adaption within glycolysis. Specifically, the ratio between the capacity of upper vs lower glycolysis is considered. By increasing the capacity of lower glycolysis over upper glycolysis, the ATP supply should be restored more quickly, without complete depletion of Pi. This effect was demonstrated by changing the size of the lower glycolysis sector in a proteome, optimized for feast/famine conditions (Figure S6). Using the energy charge, it is observed that proteomes larger lower glycolysis sector have are able to maintain a higher energy charge throughout the cycle, however, it appears that the effect on the stability of glycolysis, as indicated by G6P, FBP and Pi, is limited.


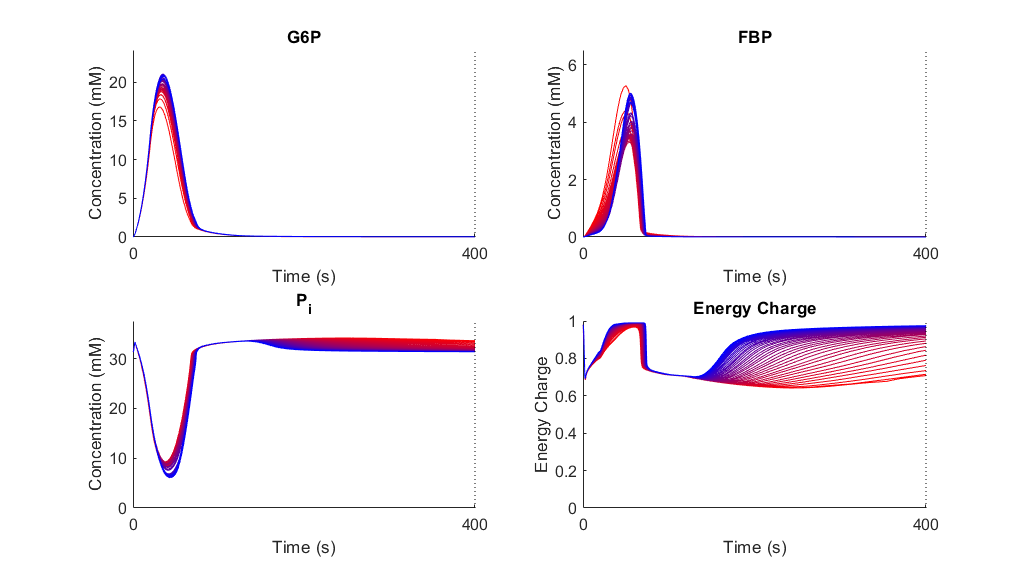


Figure S6. The extra- and intracellular metabolite profiles of proteomes with a upper glycolysis/lower glycolysis ratio between 1/10 (blue) to 1/100 (red).

# Protein categorisation

The proteome datasets are sorted in the same nine sectors that are used in the proteome-dependent kinetic model. The complete datasets are sorted based on protein names and keywords with the use of MATLAB 2020b. First, specific proteins are sorted out based on their protein name. Secondly, the remaining proteins are sorted based on keywords are sorted based on the KEGG Pathway names, the GOMF names and the GOMB names (Goffeau et al., 1996; Kanehisa et al., 2016).

Table S6. Proteins and proteins groups from KEGG sorted to their respective protein sectors defined in the model.

| Sector | Proteins/Protein groups |
| --- | --- |
| Upt | HXK, GLK, HXT |
| UGlc | PGI, FBP, PFK |
| LGlc | FBA, TPI, TDH, PGK, GPM, ENO, PYK, PCK |
| Ferm | PDC, ADH |
| ESnk | GPD, HOR, RHR |
| Resp | PYC, PDA, PDB, PDX, LPD, CIT, MDH, ACO, MLS, ICL, IDH, IDP, SDH, LCS, FUM, KGD, LPD, LAT, ALD, ACS, TCA Cycle, Oxidative phosphorylation |
| Treh | PGM, TPS, GSY, NTH, ATH, GPH |
| Grwt | Genetic Information Processing, Amino acid metabolism, Metabolism of other amino acids, Lipid metabolism, Metabolism of cofactors and vitamins, biosynthesis, Nucleotide metabolism, Cytoskeleton, Biosynthesis, Transcription, Cell cycle, DNA, Metabolism, Pentose Phosphate pathway, Ribosome, RNA, SNARE, Proteasome, Cytoskeleton, Structural, Cell wall, Translation, Biogenesis, Reproduction, Assembly, Golgi |
| Struc | HSP, HRI, PKR, PERK, GCN2, YBH, RCK, RQC, RCN, LSM, PIL, TMA, RDL, RTN, PST, STM, Not Included in Pathway or Brite, Environmental Information Processing, Cellular Processes, Signaling, Protein kinases, Protein phosphatases, Peptidases, Glycosyltransferases, Protein families: signaling and cellular processes, Organismal Systems, Stress, maintenance |

# References

de Godoy, L.M.F., Olsen, J.V., Cox, J., Nielsen, M.L., Hubner, N.C., Fröhlich, F., Walther, T.C., Mann, M., 2008. Comprehensive mass-spectrometry-based proteome quantification of haploid versus diploid yeast. Nature 455, 1251–1254. https://doi.org/10.1038/nature07341

de Vries, S., Grivell, L.A., 1988. Purification and characterization of a rotenone‐insensitive NADH: Q6 oxidoreductase from mitochondria of Saccharomyces cerevisiae. European Journal of Biochemistry 176, 377–384. https://doi.org/10.1111/j.1432-1033.1988.tb14292.x

Goffeau, A., Barrell, B.G., Bussey, H., Davis, R.W., Dujon, B., Feldmann, H., Galibert, F., Hoheisel, J.D., Jacq, C., Johnston, M., Louis, E.J., Mewes, H.W., Murakami, Y., Philippsen, P., Tettelin, H., Oliver, S.G., 1996. Life with 6000 Genes. Science 274, 546–567. https://doi.org/10.1126/science.274.5287.546

Heyland, J., Fu, J., Blank, L.M., 2009. Correlation between TCA cycle flux and glucose uptake rate during respiro-fermentative growth of Saccharomyces cerevisiae. Microbiology 155, 3827–3837. https://doi.org/10.1099/mic.0.030213-0

Illmer, P., Erlebach, C., Schinner, F., 1999. A practicable and accurate method to differentiate between intra- and extracellular water of microbial cells. FEMS Microbiology Letters 178, 135–139. https://doi.org/10.1016/S0378-1097(99)00351-1

Kanehisa, M., Sato, Y., Kawashima, M., Furumichi, M., Tanabe, M., 2016. KEGG as a reference resource for gene and protein annotation. Nucleic Acids Res 44, D457–D462. https://doi.org/10.1093/nar/gkv1070

Lamprecht, I., Schaarschmidt, B., Welge, G., 1976. Microcalorimetric investigation of the metabolism of yeasts - V. Influence of ploidy on growth and metabolism. Radiation and Environmental Biophysics 13, 57–61. https://doi.org/10.1007/BF01323624

Pronk, J.T., Steensma, H.Y., Van Dijken, J.P., 1996. Pyruvate Metabolism in Saccharomyces cerevisiae. Yeast 12, 1607–1633. https://doi.org/Doi 10.1002/(Sici)1097-0061(199612)12:16<1607::Aid-Yea70>3.0.Co;2-4

Smallbone, K., Malys, N., Messiha, H.L., Wishart, J.A., Simeonidis, E., 2011. Building a kinetic model of trehalose biosynthesis in Saccharomyces cerevisiae, 1st ed, Methods in Enzymology. Elsevier Inc. https://doi.org/10.1016/B978-0-12-385118-5.00018-9

Suarez-Mendez, C., Sousa, A., Heijnen, J., Wahl, A., 2014. Fast “Feast/Famine” Cycles for Studying Microbial Physiology Under Dynamic Conditions: A Case Study with Saccharomyces cerevisiae. Metabolites 4, 347–372. https://doi.org/10.3390/metabo4020347

Suarez-Mendez, C.A., Hanemaaijer, M., ten Pierick, A., Wolters, J.C., Heijnen, J.J., Wahl, S.A., 2016. Interaction of storage carbohydrates and other cyclic fluxes with central metabolism: A quantitative approach by non-stationary 13 C metabolic flux analysis. Metabolic Engineering Communications 3, 52–63. https://doi.org/10.1016/j.meteno.2016.01.001

Suarez-Mendez, C.A., Ras, C., Wahl, S.A., 2017. Metabolic adjustment upon repetitive substrate perturbations using dynamic 13C-tracing in yeast. Microbial Cell Factories 16. https://doi.org/10.1186/s12934-017-0778-6

Teusink, B., Passarge, J., Reijenga, C.A., Esgalhado, E., Van Der Weijden, C.C., Schepper, M., Walsh, M.C., Bakker, B.M., Van Dam, K., Westerhoff, H. V., Snoep, J.L., 2000. Can yeast glycolysis be understood terms of vitro kinetics of the constituent enzymes? Testing biochemistry. European Journal of Biochemistry 267, 5313–5329. https://doi.org/10.1046/j.1432-1327.2000.01527.x

van Heerden, J.H., Wortel, M.T., Bruggeman, F.J., Heijnen, J.J., Bollen, Y.J.M., Planque, R., Hulshof, J., O’Toole, T.G., Wahl, S.A., Teusink, B., 2014. Lost in Transition: Start-Up of Glycolysis Yields Subpopulations of Nongrowing Cells. Science 343, 1245114–1245114. https://doi.org/10.1126/science.1245114

Van Hoek, P., Van Dijken, J.P., Pronk, J.T., 1998. Effect of Specific Growth Rate on Fermentative Capacity of Baker’s Yeast. Appl Environ Microbiol 64, 4226–4233. https://doi.org/10.1128/AEM.64.11.4226-4233.1998

Vos, T., Hakkaart, X.D.V., Hulster, E.A.F., Maris, A.J.A., Pronk, J.T., Daran-Lapujade, P., 2016. Maintenance-energy requirements and robustness of Saccharomyces cerevisiae at aerobic near-zero specific growth rates. Microbial Cell Factories 15, 1–20. https://doi.org/10.1186/s12934-016-0501-z
